# Efficacy and safety of Danggui Niantong Decoction in patients with gout: a systematic review and meta-analysis

**DOI:** 10.3389/fphar.2023.1168863

**Published:** 2023-07-26

**Authors:** Sihan Peng, Jing Tian, Luchang Jin, Hongyan Wang, Chunguang Xie, Jie Zheng, Linfeng Liu, Jun Cao, Wen Zhang, Xiangeng Zhang

**Affiliations:** ^1^ TCM Regulating Metabolic Diseases Key Laboratory of Sichuan Province, Hospital of Chengdu University of Traditional Chinese Medicine, Chengdu, Sichuan, China; ^2^ Chengdu University of Traditional Chinese Medicine, Chengdu, Sichuan, China; ^3^ Huaxi Securities Co., Ltd., Chengdu, Sichuan, China; ^4^ Sichuan Nursing Vocational College, Chengdu, Sichuan, China; ^5^ College of Basic Medical Sciences, Air Force Medical University, Xian, Shaanxi, China

**Keywords:** Danggui Niantong Decoction, gout, efficacy, safety, systematic review, meta-analysis

## Abstract

**Background:** This study aims to evaluate the efficacy and safety of Danggui Niantong Decoction (DGNT) systematically on gout treating.

**Methods:** This study was registered in PROSPERO, and the registration number was CRD42021271607. By the end of December, 2022, literature research was conducted among eight electronic databases. Main results of this study were blood uric acid (BUA) and Creactive protein (CRP). Secondary outcomes were erythrocyte sedimentation rate (ESR), serum creatinine (Scr), urinary protein quantified at 24 h (Upro), and interleukin-8 (IL-8). Study screening, data collection, as well as quality assessment were performed by two reviewers independently, and analysis was completed using Stata (SE15.0) and Review Manager (5.4).

**Results:** A total number of 13 studies were included in our meta-analysis (*n* = 1,094 participants). Results showed DGNT combined with conventional western medicine (CWM) was more effective than WM alone in BUA (weighted mean differences (WMD) = −3.49, 95% confidence interval (CI) [−50.36, −32.59], *p* = 0.000), CRP (WMD = −41.48, 95% CI [−4.32, −2.66], *p* = 0.017), ESR (WMD = −6.23, 95% CI [−9.28, −3.17], *p* = 0.019), Scr (WMD = −18.64, 95% CI [−23.09, −14.19], *p* = 0.001), Upro (WMD = −0.72, 95% CI [−0.91, −0.53], *p* = 0.000), and IL-8 (WMD = −4.77, 95% CI [−11.48, 1.94], *p* = 0.000). None of the adverse effects noted were severe, and no life-threatening event was reported.

**Conclusion:** This study shows that DGNT combined with CWM seems to have an effective clinical therapeutic potential. In addition, it also provides a scientific basis for better clinical application of DGNT in the future.

**Systematic Review Registration:** https://www.crd.york.ac.uk/prospero/display_record.php?ID=CRD42021271607; Identifier: PROSPERO, CRD42021271607.

## 1 Introduction

Gout is a form of arthritis caused by accumulation of uric acid crystals in the body (Yip and Berman, 2021). Due to acute inflammation of the affected joint or joint, asymptomatic periods of indefinite length is one of the characteristics of gout attack, and it also has long been associated with various dietary induced and environmental exposures (Caution et al., 2019; Wu et al., 2022; Yang et al., 2022). Hyperuricemia has become the second largest metabolic disease after diabetes (Liu et al., 2021) due to improvement in the standard of living and change in diet structure. Additionally, which is an independent risk factor for the progression of cardiovascular diseases, kidney diseases, and diabetes. The incidence of gouty nephropathy, which is a kidney injury caused by hyperuricemia, is increasing (Liu et al., 2017). The initial stage of gouty nephropathy is characterized by an elevated blood uric acid (BUA), nocturia, proteinuria, edema, and normal or slightly elevated creatinine (CRE) (Borghi et al., 2020). Moreover, it may progress to end-stage renal disease if the condition cannot get controlled (Yanai et al., 2021). Gout affects approximately 9.2 million people in the U.S. (about 3.9% of the adult population) (Chen-Xu et al., 2019), and rather common among male and elderly people (Yip and Berman, 2021). It is one of the most common inflammatory arthritis for men greater than 40 years old. The reported prevalence of gout ranges from 0.1%–10% (Stamp et al., 2021). According to the latest Global Burden of Disease (GBD) estimate, gout affected 41 million people worldwide, and the incidence kept rising (Safiri et al., 2020). The common complications of gout aggravate the burden of gout, including hypertension (75%), chronic kidney disease (CKD) (70%), obesity (53%), and cardiovascular diseases (CVDs) (10%–14%), all of which are related to high morbidity and risk of death (Danve et al., 2021). It greatly harms the global public health.

Anti-inflammatory therapy is usually considered as the first line treatment for gout, and colchicine, nonsteroidal anti-inflammatory drugs, corticosteroids were commonly used (FitzGerald et al., 2020; McKenzie et al., 2021). However, there is no perfect treatment for gout, and even the most effective treatment options have their problems. Moreover, the prognosis of patients was still poor. Although uric acid could be reduced by western medicine and further alleviate the kidney damage, patients with severe hepatic and renal insufficiency, and cardiovascular and cerebrovascular diseases still needs to take treatment with caution.

Additionally, long-term use of certain drugs may bring adverse reactions, as well as high recurrence rate after withdrawal (Zhou Ximou and He, 2021). Consequently, reliable alternative strategies for the treatment of gout are urgently needed.

Traditional Chinese medicine (TCM) is one of the major systems of complementary and alternative medicine that have been developed in China for thousands of years. It has a long history on treating gout, which can provide individualized treatment for patients (Li Wen and Ke, 2020). DGNT is a famous Chinese prescription which was recorded for the first time by Zhang Yuansu in the “Yixue Qiyuan" (Yuansu, 2007). Recently, increasing clinical evidence indicate that DGNT significantly ameliorates the clinical symptoms of gout, and hence improves the pathological changes in the joints of gout patients (Lu Junguang and Zheng, 2021). Therefore, DGNT is considered to be a possible effective method to treat gout with TCM.

Recent studies have shown that DGNT is effective in treating gout since it reduces the incidence of gouty nephropathy by lowering uric acid levels (Wei Ailing et al., 2020; Sike, 2022). DGNT was able to reduce BUA content, endothelin −1 (ET-1), blood urea nitrogen (BUN) and CRE, and it was able to protect renal endothelial cells and glomerular blood vessels (Yao Lusha, 2018; Li Yuxuan and Yu, 2019). The study has shown that DGNT has multiple targets in the treatment of acute gouty arthritis (AGA), and it may play a preventive role by regulating Akt/Bax/Bcl-2 pathway and promoting apoptosis in the mitochondrial pathway (Cai Yisi et al., 2022). Nonetheless, the current studies are mostly single-center and small-sample studies, and have different research designs. Therefore, we aimed to evaluate the efficacy and safety of DGNT systematically, and provide a basis for its use in clinical practice for the treatment of gout through evidence-based medicine methods.

## 2 Methods

### 2.1 Search strategy and selection criteria

This systematic review and meta-analysis are in accordance with the Preferred Reporting Items for Systematic Reviews and Meta-Analyses (PRISMA) Statement. Additionally, the review was registered at PROSPERO (**CRD42021271607**).

By the end of December 2022, eight electronic databases were explored, including Web of Science, Cochrane Library, PubMed, Embase, China National Knowledge Infrastructure (CNKI), Wanfang Database, VIP information resource integration service platform (cqvip), and China Biology Medicine Disc (Sino Med), in order to find more literature about DNGT on gout treating. We included all potentially eligible studies for review, irrespective of the primary outcome or language. Additionally, we did a manual search using the reference lists of key articles published in English. We included all randomized controlled trials (RCTs) that evaluated the efficacy and safety of Danggui Niantong decoction in gout patients. The keywords used in these searches were as follow: “Gout”, “Gouts”, “Chondrocalcinosis”, “Danggui Niantong decoction”, “Danggui Niantong”, and “modified Danggui Niantong decoction”. See the supplementary document for the comprehensive search strategy of the database (Supplementary Table S1).

### 2.2 Inclusion and exclusion criteria

According to the root “PICOS” principle, it was screened. These studies are considered eligible for inclusion in the analysis if they meet all the following criteria and circumstances: 1) Population: patients must be greater than or equal to 18 years old (pregnant women are excluded), and met the latest diagnostic criteria for gout (Neilson et al., 2022). 2) Control: CWM was used in the control group. 3) Intervention: DGNT combined with CWM was used in the intervention group. 4) Outcome: Main results of this study were BUA and C-reactive protein (CRP). The secondary outcomes were erythrocyte sedimentation rate (ESR), serum creatinine (Scr), urinary protein quantified at 24 h (Upro), and interleukin-8 (IL-8). 5) Study design: RCT.

The exclusion criteria were as follows: 1) Non-RCTs, such as case reports and reviews. 2) Besides DGNT and conventional treatment, there are other TCM interventions, including herbal medicine, acupuncture, or acupoint injection therapy. 3) Duplicate publications, animal experiments, and incomplete or unavailable data.

### 2.3 Study selection and data extraction

At first, two independent investigators (SHP, JT) reviewed the titles and abstracts of each study, and studies that satisfied the inclusion criteria were retrieved for full-text assessment. Then, the studies selected for detailed analysis and data extraction were analyzed by SHP and JT. If there were any discrepancies or disagreements, they were resolved by a third investigator (XGZ). Endnote 20 was used to manage literature. Relevant data were independently extracted from eligible studies by three reviewers (JC, LFL and WZ), including authors, publication time, age, gender, course of disease, total number of patients, intervention methods and duration, mean and standard deviation (SD) of BUA, CRP, ESR, Scr, Upro, and IL-8, using standardized extraction forms. The independent reviewers also assessed the risk of bias according to the PRISMA recommendations.

### 2.4 Risk of bias assessment

The two reviewers (SHP, JT) independently evaluated the quality of the methods of the trials according to the Cochrane manual. Items such as random sequence generation, allocation concealment, blinding of participants and personnel, the blindness of outcome assessments, incomplete outcome data, selective outcome reporting, and other biases should be contained. The results were assessed as ‘low,’ ‘high,’ or ‘unclear’. Each disagreement was discussed and resolved with the third investigator (XGZ).

### 2.5 Data synthesis and statistical analysis

To analyze and evaluate the effect of DGNT on patients with gout we assessed BUA, CRP, ESR, Upro, Scr, and IL-8 as continuous variables. All data were weighted and merged, with the weighted mean difference (WMD) and mean difference (MD) as effect indicators, while the point estimates and 95% confidence interval (CI) for the joint effect were evaluated. A *p*-value of <0.05 was regarded as statistically significant.

To assess the reliability and stability of the combined results, we performed meta-analyses and pre-planned sensitivity analyses for each outcome. (see Supplementary Figure S2). This comparison is the most important clinical question pertaining to the effect of DGNT on gout, and reduced the heterogeneity of the treatment changes in outcomes from the intervention group seen among the overall analysis.

The heterogeneity was assessed using the I^2^ value. If the I^2^ values were greater than 50%, a significant statistical heterogeneity was indicated (Sun et al., 2010). To find potential reasons of the heterogeneity, we performed subgroup analyses, and focused on the following factors: Complications (having complications or not), intervention duration (1W, 2W, 4W, or 12W), different intervention treatments (using colchicine or not), and different ages (≥50y or <50y).

We assessed the possibility of a publication bias by constructing a funnel plot of the effect size of trial against the standard error (Supplementary Figure S3). We assessed funnel plot asymmetry by using Egger’s tests, and defined significant publication bias as a *p*-value of <0.1. The trim-and-fill computation was used to estimate the effect of publication bias on the interpretation of the results (Duval and Tweedie, 2000). The Cochran Q test was used to assess the heterogeneity between studies (Higgins et al., 2003). We also conducted I^2^ testing to assess the magnitude of the heterogeneity between studies, with values >50% indicating moderate-to-high heterogeneity (Sidik and Jonkman, 2022). Stata (version 15.0) and Review Manager (version 5.4) were used for all statistical analyses.

## 3 Results

### 3.1 Literature selection

**FIGURE 1:**
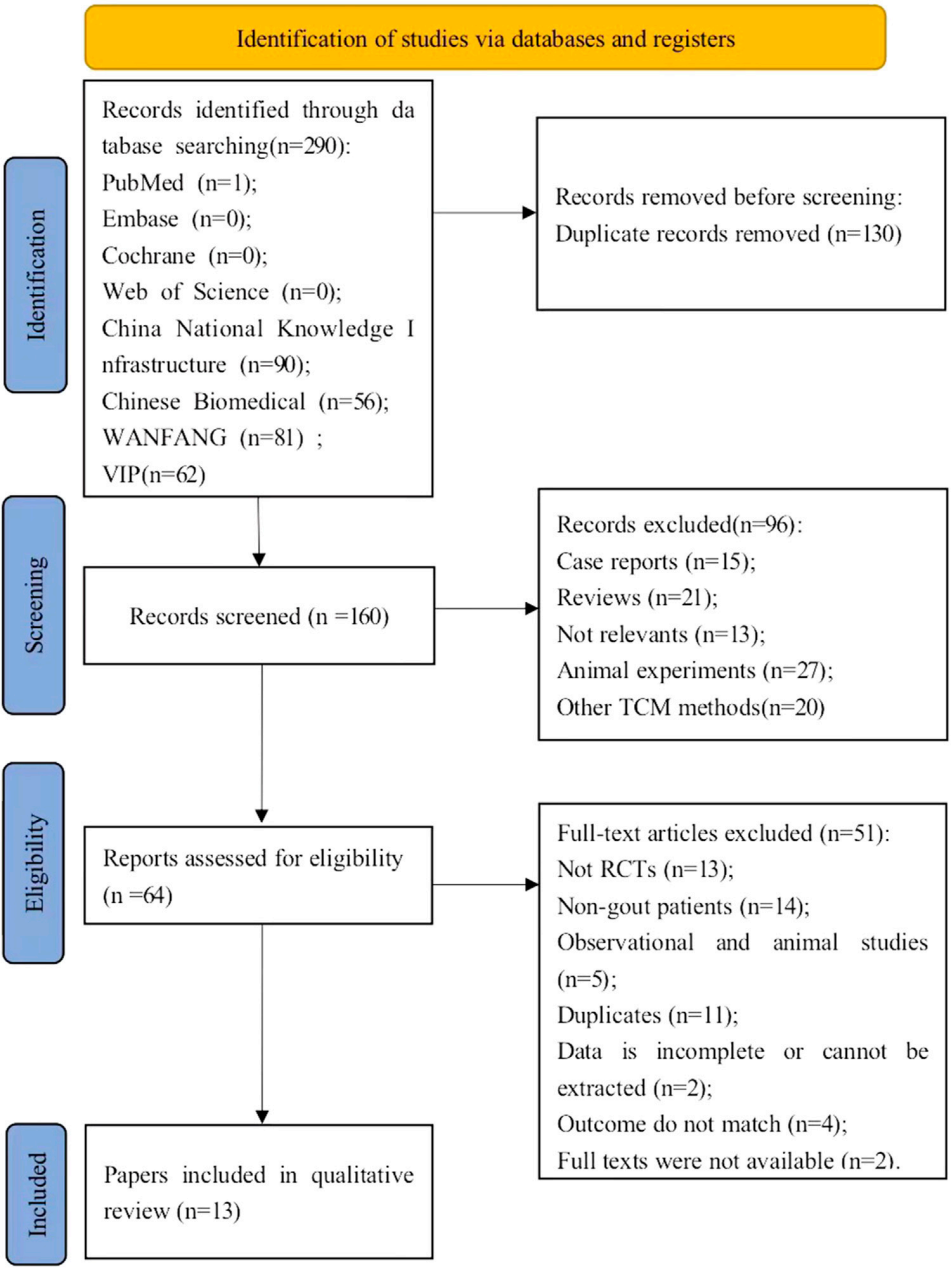
PRISMA flow chart for literature search.

In all, we preliminarily collected 290 potentially relevant articles from 8 databases. First, duplicate articles were removed, and remained 160 articles for title and abstract screening. Among them, 96 articles were excluded because they did not meet the inclusion criteria, such as case reports, reviews, studies unrelated to the study subject, animal experiments, the presence of other TCM methods. After intensive reading of the full texts of the remaining 64 articles, 51 articles were excluded for the following reasons: (1)Not RCTs (n = 13); (2)Non-gout patients (n = 14); (3)observational and animal studies (n = 5); 4) duplicate studies: there is duplication in study author, study center, study design, study data, and results (n = 11); 5) the data is incomplete or cannot be extracted (n = 2); 6) the outcome do not match (n = 4); 7) full texts were not available (n = 2). Ultimately, we retained 13 studies (Luo Jinlin and Luo, 2012; Shang, 2012; Chunxiao, 2015; Huang Juntao and Ge, 2016; Junhong, 2018; Yue Zhao, 2019; Dai Fengxiang, 2020; Guoqiang, 2020; Yonghui, 2020; Chenjia, 2021; Guiyang, 2021; Lei Xiaojun, 2021; Lu Junguang and Zheng, 2021) in our meta-analysis. The process of selection of articles is depicted in Figure 1.

### 3.2 Study characteristics

A total of 1,094 patients (552 in treatment group and 542 in control group) from the 13 studies were enrolled in this study. All the studies that we included were RCTs conducted in China, and the publishing time ranged from 2012–2021. Meta-analysis is included in the research, the experimental group used DGNT alone or combined with conventional treatment, while the control group used conventional treatment. The treatment duration lasted at least 1 week (Dai Fengxiang, 2020; Yonghui, 2020; Chenjia, 2021; Guiyang, 2021; Lu Junguang and ZHeng, 2021) to 12 weeks (Shang, 2012), and the baseline characteristics and details of the included studies are shown in Table 1. By referring to the “type A extract” of ConPhyMP consensus statement (Heinrich et al., 2022), we compiled a summary table describing the botanical drug components and how it was reported in the original research. As a type A extract, DGNT is composed of 15 botanical drugs and functions to clear heat away, promote diuresis and relieve pain. DGNT was the basic prescription in all studies, and botanical drugs, such as *Angelica sinensis* (Oliv.) Diels [Umbelliferae; Angelicae Sinensis Radix](dāng guī), *Notopterygium incisum* Ting ex H. T. Chang [Umbelliferae; Rhizoma Notopterygii](qiāng huó), *Atractylodes lancea* (Thunb.) DC. [Asteraceae; Atractylodis rhizoma](cāng zhú), *Atractylodes macrocephala* Koidz. [Asteraceae: Atractylodis macrocephalaerhizoma](bái zhú), *Artemisia capillaris* (Thunb.)DC. [Compositae; Artemisia capillaris](yīn chén), *Polyporus umbellatus* (Pers)Fr. [polyporaceae; Polyporus] (zhū líng), *Alisma orientalis* (Sam.)Juzep. [Alismataceae; Alismatis Rhizoma](zé xiè), *Scutellaria baicalensis* Georgi. [Labiatae; Scutellariae Radix](huáng qín), *Anemarrhena asphodeloides* Bunge [Liliaceae; Anemarrhenae Rhizoma](zhī mǔ), *Saposhnikovia divaricata* (Trucz.) Schischk. [Umbelliferae; Radix Saposhnikoviae](fáng fēng), *Sophora flavescens* Alt. [Leguminosae; Sophorae Flavescentis Radix](kǔ shēn), *Cimicifuga foetida* L. [Ranunculaceae; Cimicifugae Rhizoma](shēng má), *Radix Puerariae* Lobatae. [Leguminosae; Radix Puerariae](gě gēn), *Panax ginseng* C. A. Mey. [Araliaceae; Ginseng Radix Et Rhizoma](rén shēn), and *Glycyrrhiza uralensis* Fisch. ex DC. (Fabaceae: Glycyrrhizae radix et rhizoma](gān cǎo), were added or subtracted according to syndrome differentiation. The composition of the prescriptions is shown in Supplementary Table S2.

**TABLE 1:** Characteristics of the included studies.

Study	Age (Y)		Course of a disease		Gender (M/F)		Complications	Study sample size (T/C)	Intervention		Duration	Outcomes
	T	C	T	C	T	C			T	C		
Chenjia (2021)	51.75 ± 18.60	46.65 ± 14.84	6.95 ± 4.81Y	7.05 ± 4.07Y	18/2	18/2	Hypertension, diabetes, hyperlipidemia	20/20	DGNT and oral diclofenac sodium sustained-release capsule, febuxostat	Oral diclofenac sodium sustained-release capsules and febuxostat	1W	①②③④
Dai Fengxiang et al. (2020)	46.75 ± 13.27	47.90 ± 12.54	44.64 ± 7.7 m	41.62 ± 9.19 m	60/5	61/4	NR	65/65	DGNT and oral administration of Celebrex, sodium bicarbonate tablets	Oral Celebrex and sodium bicarbonate tablets	1W	①②③
Huang Juntao and Ge (2016)	52.70 ± 2.90	51.30 ± 1.60	5.10 ± 2.90Y	5.30 ± 2.10Y	39/11	38/12	NR	50/50	DGNT and oral colchicine	Oral colchicine	4W	②⑥
Chunxiao (2015)	43.60 ± 5.10	42.90 ± 5.30	57.40 ± 7.20 m	56.80 ± 8.50 m	30/16	28/18	NR	46/46	DGNT and oral benzbromarone tablets	Oral benzbromarone tablets	2W	⑥
Lu Junguang and Zheng (2021)	43.61 ± 7.30	44.06 ± 7.10	1.04 ± 0.40Y	1.11 ± 0.40Y	32/9	31/8	NR	41/39	DGNT and modified diclofenac sodium enteric-coated tablets	Diclofenac sodium enteric-coated tablets	1W	①
Luo Jinlin and Luo (2012)	47.20 ± 5.40	47.00 ± 5.10	45.20 ± 9.60 m	45.70 ± 9.10 m	27/9	26/8	NR	36/34	DGNT and oral colchicine	Oral colchicine	2W	②⑥
Yonghui (2020)	62.00	61.00	5.60 ± 1.70D	6.90 ± 2.10D	17/3	18/2	NR	20/20	DGNT and colchicine tablets, sodium bicarbonate tablets	Colchicine tablets, sodium bicarbonate tablets	1W	①③
Guiyang (2021)	57.02 ± 6.08	56.97 ± 6.13	9.01 ± 1.39Y	8.92 ± 1.33Y	38/8	37/9	NR	46/46	DGNT and addition and subtraction of Celecoxib	Celecoxib	1W	①②
Sun et al. (2010)	49.81 ± 7.22	49.02 ± 6.83	NR	NR	23/17	24/16	NR	40/40	DGNT and oral allopurinol tablets, sodium bicarbonate tablets	Oral allopurinol tablets and sodium bicarbonate tablets	4W	①④⑤
Yue Zhao (2019)	48.89 ± 6.25	49.94 ± 6.10	9.79 ± 1.42Y	9.76 ± 1.33Y	45/19	44/20	Hypertension, diabetes, hyperlipidemia, chronic renal insufficiency, lithangiuria	64/64	DGNT and febuxostat tablet	On the basis of symptomatic treatment, febuxostat tablets were added.	2W	①④⑤
Liu (2017)	49.90 ± 3.30	49.50 ± 3.60	NR	NR	17/13	16/14	NR	30/30	DGNT and colchicine, prednisone, allopurinol, bupleurum Modified Sita, Probenecid	Oral colchicine, prednisone, allopurinol, and fenbu Sita, probenecid *(continued)*	2W	①④⑤
Lei Xiaojun (2021)	50.36 ± 5.98	51.24 ± 5.68	11.03 ± 2.98Y	10.12 ± 2.65Y	22/21	22/21	NR	43/43	DGNT and allopurinol tablets, sodium bicarbonate tablets	Oral allopurinol tablets and sodium bicarbonate tablets	2W	①④⑤
Huang (2012)	49.70 ± 11.90	49.20 ± 11.80	7.90 ± 4.00Y	7.80 ± 3.90Y	38/13	36/9	Hypertension, diabetes, hyperlipidemia, chronic renal insufficiency, lithangiuria	51/51	DGNT and allopurinol tablets, sodium bicarbonate tablets	On the basis of symptomatic treatment, allopurinol tablets and sodium bicarbonate tablets were taken orally.	12W	①④⑤

Abbreviations: T, treatment group; C, control group; F, female; M, male; NR, not reported; Y, year; m, month; W, week; D, day; ①:BUA, ②:CRP, ③:ESR, ④:Scr, ⑤:Upro, ⑥:IL-8.

### 3.3 Risk of bias assessment

**FIGURE 2:**
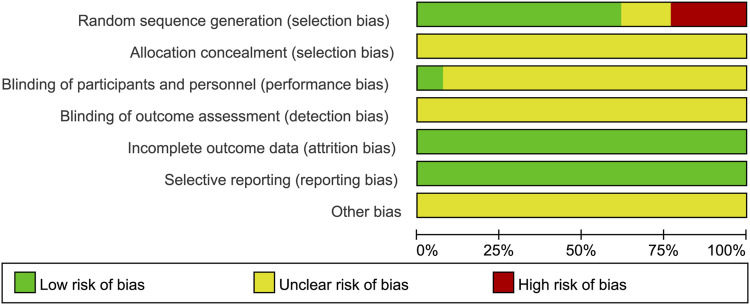
Risk of bias graph.

**FIGURE 3:**
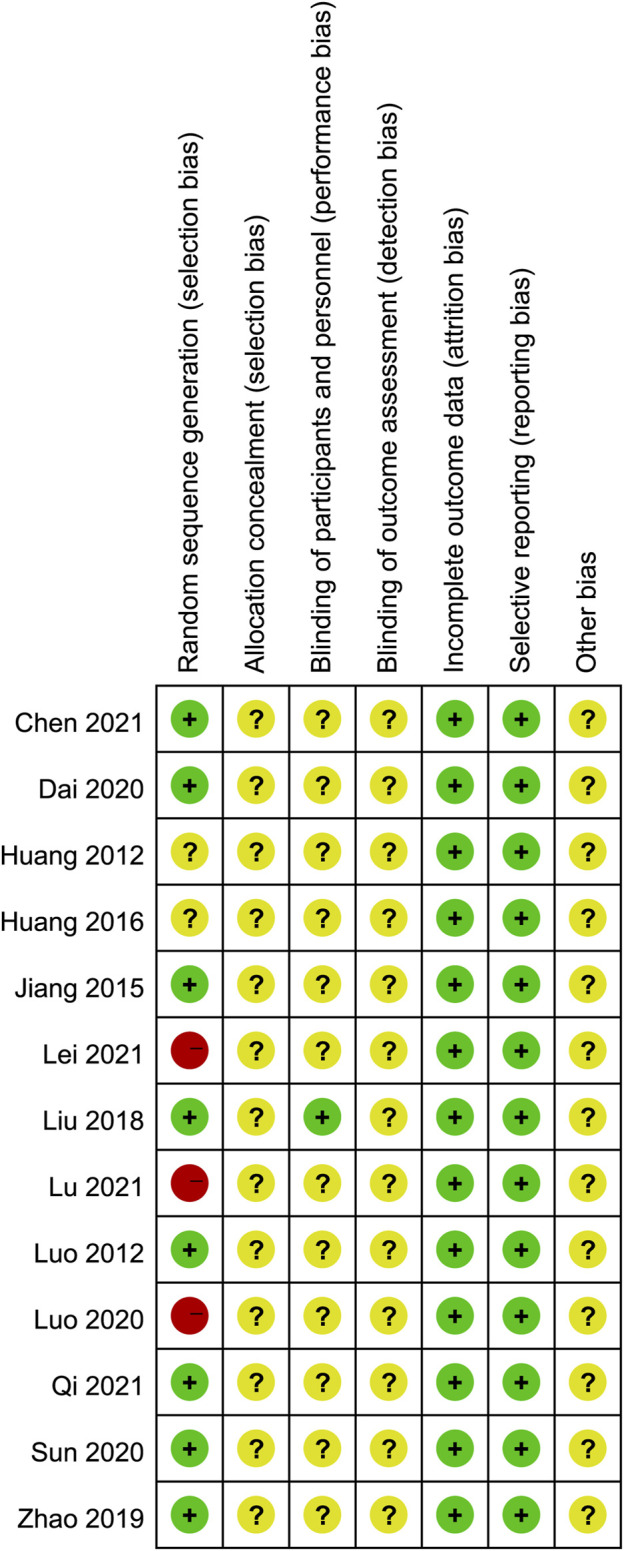
Risk of bias summary.

The results of the risk of bias assessment are shown in Figure 2 and Figure 3. In all, most of the trials included had low to medium qualities, and 8 studies (Luo Jinlin and Luo, 2012; Chunxiao, 2015; Junhong, 2018; Yue Zhao, 2019; Dai Fengxiang, 2020; Guoqiang, 2020; Chenjia, 2021; Guiyang, 2021) were randomly assigned, which used the method of random number table. However, none of the included studies described the method of randomization hidden. Only one study (Junhong, 2018) clearly pointed out the implementation of single blindness, while other studies have not yet reported whether to implement the blind method, hence it is classified as “unclear risk.” Evaluation of the intervention effectiveness was performed by comparing the objective outcome indicators between the treatment and control groups, which was thought to reduce the bias caused by blinding method. However, for the other biases, all studies provided insufficient information to identify other significant risks of bias existing, and therefore they were assessed as “unclear risk.”

### 3.4 Outcomes

We analyzed and compared the efficacy and safety of DGNT for the treatment of gout with the conventional treatment, with no restriction on treatment history. The outcomes assessed were as follows.

#### 3.4.1 Primary outcome measures

##### 3.4.1.1 Blood uric acid (BUA)

**FIGURE 4:**
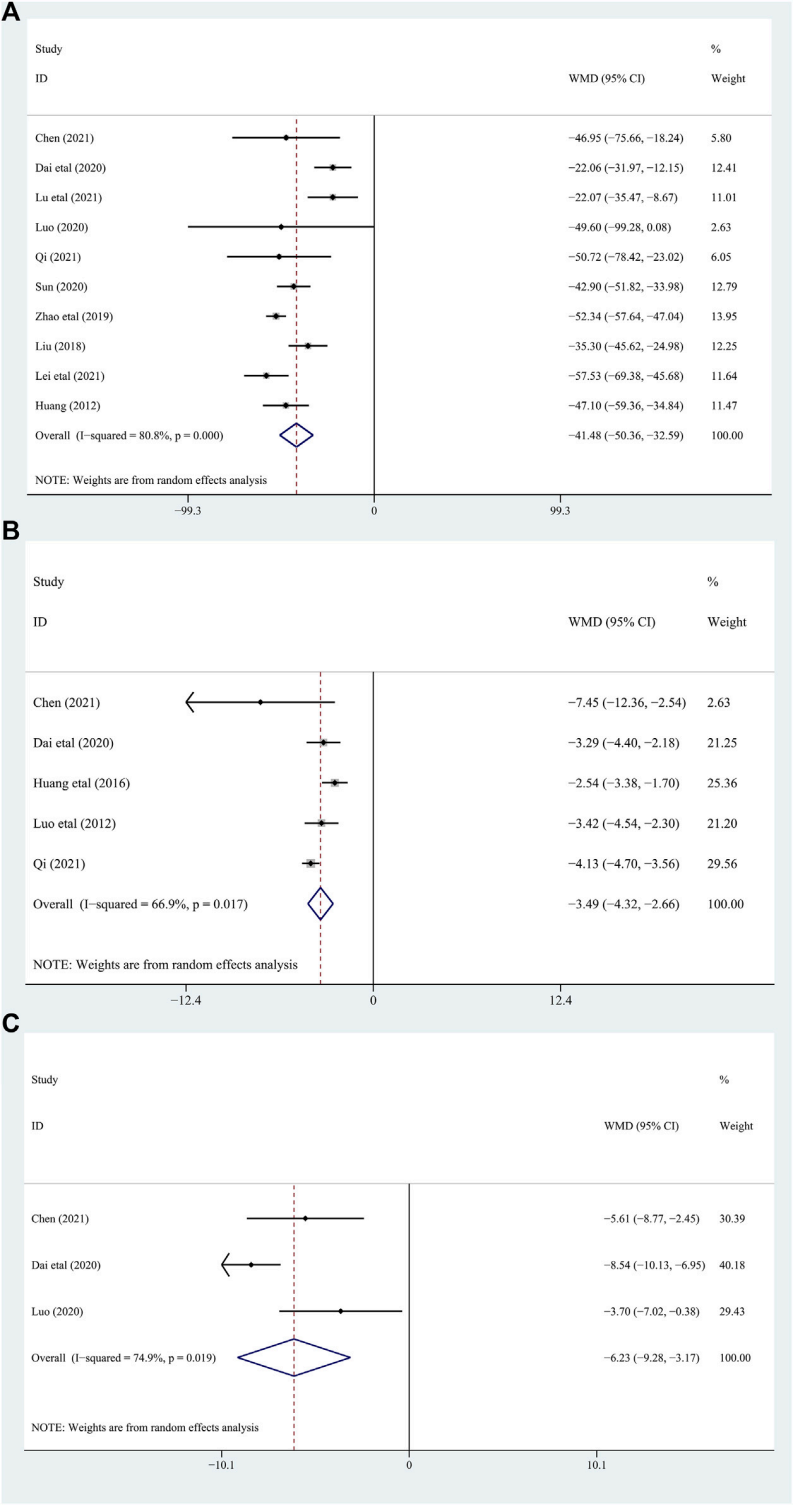
Forest plot [**(A)**: BUA, **(B)** CRP, **(C)** ESR].

**FIGURE 5:**
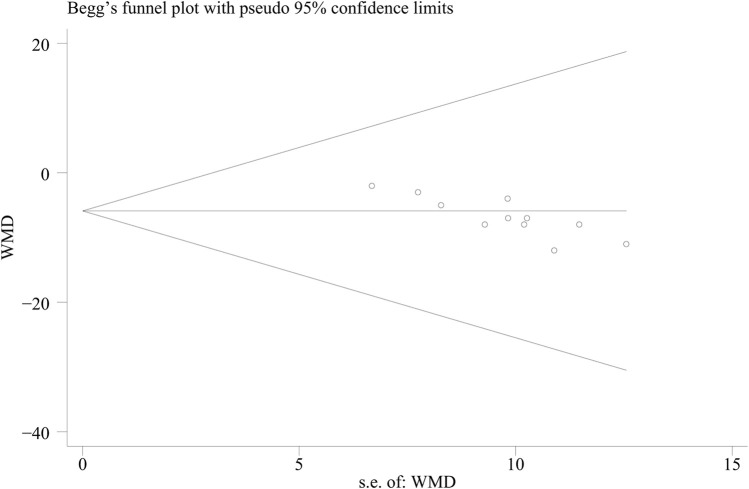
Publication bias (BUA).

Among the included studies, 10 studies (Shang, 2012; Junhong, 2018; Yue Zhao, 2019; Dai Fengxiang, 2020; Guoqiang, 2020; Yonghui, 2020; Chenjia, 2021; Guiyang, 2021; Lei Xiaojun, 2021; Lu Junguang and Zheng, 2021) with 832 gout patients involved, and provided the data of BUA levels before and after intervention of the treatment and control groups. After intervention, the BUA levels in the DGNT group significantly decreased [WMD = −41.48; 95% CI (−50.36, −32.59); *p* = 0.000, random effects model; see Figure 4]. Significant heterogeneity exists (I^2^ = 80.8%; see Figure 4). Subgroup analysis showed that there were a significant difference in whether there were complications (*p* = 0.03), and no significant difference between different treatment duration (*p* = 0.60) and different intervention treatments (*p* = 0.39) was observed (Table 2; Supplementary Figure S1). Furthermore, the Egger’s test indicated no obvious publication bias in this analysis (see Figure 5).

**TABLE 2:** Subgroup analysis for outcomes.

	Number of comparisons	Results	*p*-value for overall effect	I^2^	*p*-value for subgroup difference
BUA		WMD(95%CI)			
All comparisons	10	−41.47 [-50.36,-32.58]	<0.00001	81%	
Complications					0.03
Have complications	3	−51.39 [-56.19,-46.59]	<0.00001	0%	
No complications	7	−37.93 [-49.04,-26.82]	<0.00001	79%	
Duration(W)					0.6
Less than 4 weeks	8	−40.59 [-52.54,-28.63]	<0.00001	85%	
More than 4 weeks	2	−44.35 [-51.57,-37.14]	<0.00001	0%	
Different intervention treatments					0.39
Colchicine	2	−35.89 [-46.00,-25.78]	<0.00001	0%	
No colchicine	8	−42.14 [-52.33,-31.94]	<0.00001	84%	
CRP					
All comparisons	5	−3.49 [-4.32,-2.66]	<0.00001	67%	
Duration(W)					0.02
Less than 4 weeks	4	−3.81 [-4.48,-3.13]	<0.00001	33%	
More than 4 weeks	1	−2.54 [-3.38,-1.70]	<0.00001	No	
Different intervention treatments					0.009
Colchicine	2	−2.86 [-3.53,-2.19]	<.00001	34%	
No colchicine	3	−3.58 [-3.99,-3.18]	<.00001	45%	
ESR					
All comparisons	3	−6.225 [-9.277,-3.173]	<.00001	74.90%	
Different intervention treatments					0.09
Colchicine	1	−3.70 [-7.02,-0.38]	0.03	No	
No colchicine	2	−7.41 [-10.20,-4.61]	<0.00001	62%	
Age					0.007
<50y	1	−8.54 [-10.13,-6.95]	<0.00001	No	
≥50y	2	−4.70 [-6.99,-2.41]	<0.0001	0%	
Scr					
All comparisons	6	−18.96 [-23.12,-14.81]	0.002	74%	
Duration(W)					0.45
Less than 4 weeks	4	−17.56 [-24.20,-10.93]	<0.00001	84%	
More than 4 weeks	2	−20.50 [-24.33,-16.68]	<0.00001	0%	
Different intervention treatments					0.52
Colchicine	1	−20.70 [-25.84,-15.56]	<0.00001	No	
No colchicine	5	−18.30 [-23.48,-13.13]	<0.00001	79%	
Complications					0.33
Have complications	3	−15.44 [-25.82,-5.06]	0.004	89%	
No complications	3	−20.77 [-23.84,-17.69]	<0.00001	0%	
Upro					
All comparisons	5	−0.72 [-0.91,-0.53]	<0.00001	93%	
Duration(W)					0.15
Less than 4 weeks	3	−0.84 [-1.11,-0.57]	<0.00001	95%	
More than 4 weeks	2	−0.55 [-0.84,-0.25]	0.0007	91%	
Different intervention treatments					0.28
Colchicine	1	−0.60 [-0.68,-0.52]	<0.00001	No	
No colchicine	4	−0.76 [-1.03,-0.48]	<0.00001	95%	
Complications					0.19
Have complications	2	−0.55 [-0.85,-0.26]	<0.0001	95%	
No complications	3	−0.85 [-1.18,-0.52]	<0.00001	95%	
IL-8					
All comparisons	3	−4.77 [-11.48,1.94]	0.0001	89%	
Different intervention treatments					<0.0001
Colchicine	2	−7.99 [-11.85,-4.13]	<0.0001	0.00%	
No colchicine	1	0.22 [0.16,0.28]	<0.00001	No	
Duration(W)					0.16
Less than 4 weeks	2	−2.70 [-9.52,4.12]	0.44	83%	
More than 4 weeks	1	−8.91 [-14.09,-3.73]	0.0008	No	

##### 3.4.1.2 C-reactive protein (CRP)

Five studies (Luo Jinlin and Luo, 2012; Huang Juntao and Ge, 2016; Dai Fengxiang, 2020; Chenjia, 2021; Guiyang, 2021) involving 432 patients with gout explored the CRP levels in the treatment and control groups. The results of these studies showed that DGNT significantly reduces CRP [WMD = −3.49; 95% CI (−4.32, −2.66); *p* = 0.017, random effects model; see Figure 4]. Significant heterogeneity exists (I^2^ = 66.9%; see Figure 4). According to the subgroup analyses based on different duration and different intervention treatments, there are significant differences in the intervention effects among the groups (*p* for interaction = 0.02, 0.009, respectively) (Table 2; Supplementary Figure S1), and significant heterogeneity was still observed. Despite statistical heterogeneity between these studies, the results of all studies showed that the use of DGNT therapy is beneficial for CRP management.

#### 3.4.2 Secondary outcome measures

##### 3.4.2.1 Erythrocyte sedimentation rate (ESR)

Only 3 studies (Dai Fengxiang, 2020; Yonghui, 2020; Chenjia, 2021) including 210 patients evaluated the change in ESR. The results indicated that DGNT significantly reduces the ESR levels compared with conventional western medicine (CWM) group [WMD = −6.23; 95% CI (−9.28, −3.17); *p* = 0.019, random effects model; see Figure 4]. There was a significant heterogeneity observed (I^2^ = 74.9%; see Figure 4). According to the subgroup analysis of age and different intervention treatments, the intervention effect between the two groups was significantly different (*p* for interaction = 0.007, 0.09, respectively) (Table 2; Supplementary Figure S1).

##### 3.4.2.2 Serum creatinine (Scr)

**FIGURE 6:**
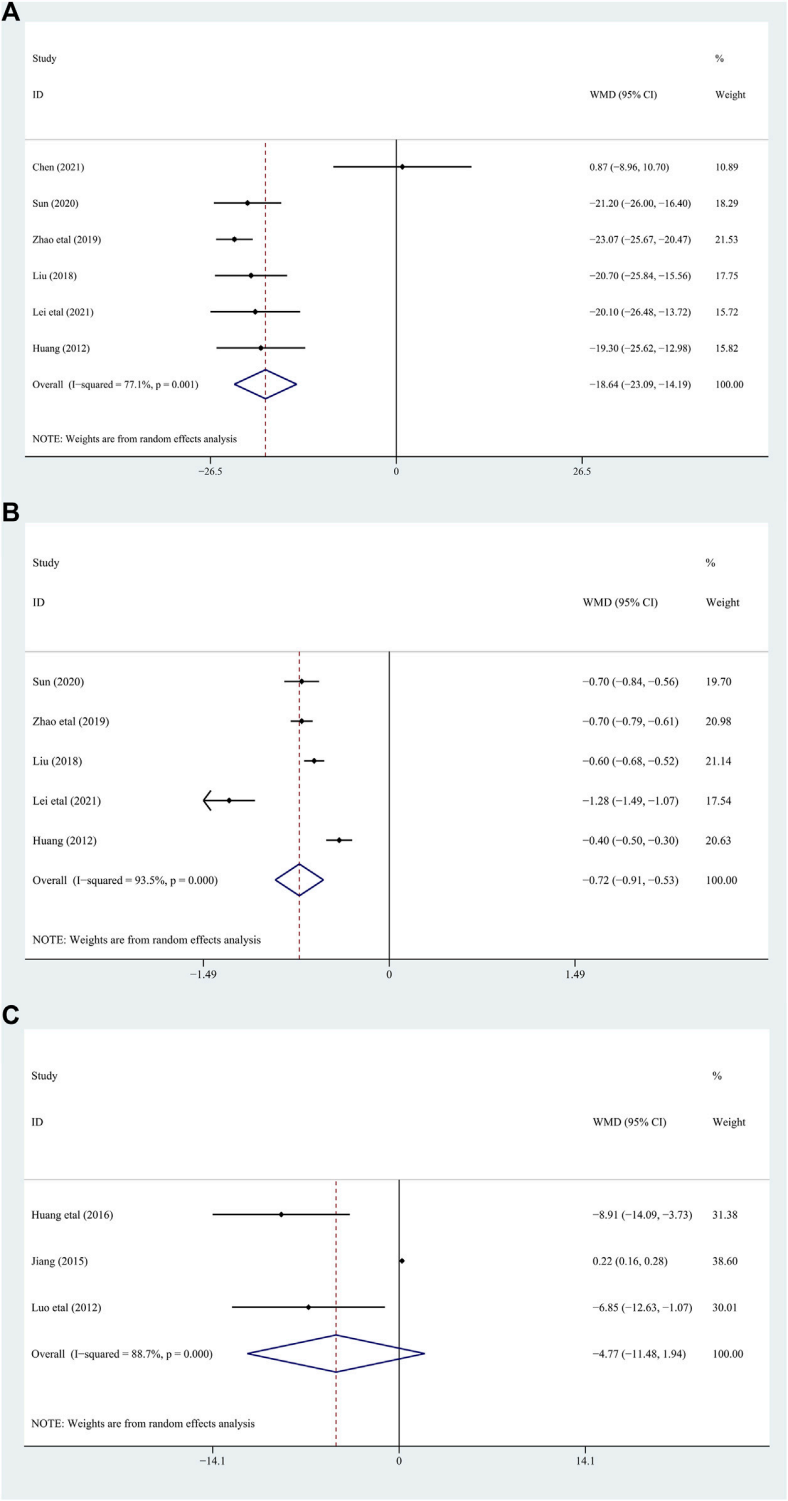
Forest plot [**(A)**: Scr, **(B)** Upro, **(C)** IL-8].

6 studies (Shang, 2012; Junhong, 2018; Yue Zhao, 2019; Guoqiang, 2020; Chenjia, 2021; Lei Xiaojun, 2021) including 490 patients assessed Scr as a biomarker. There were significant differences between the two groups [WMD = −18.64; 95% CI (−23.09, −14.19); *p* = 0.001, random effects model; see Figure 6]. The heterogeneity was significant. (I^2^ = 77.1%; see Figure 6). In subgroup analyses, there was no significant difference between the subgroups of different duration, complications, and different intervention treatments (*p* for interaction = 0.45,0.33, and 0.52, respectively) (Table 2; Supplementary Figure S1).

##### 3.4.2.3 Urinary protein was quantified at 24 h (Upro)

Five studies (Shang, 2012; Junhong, 2018; Yue Zhao, 2019; Guoqiang, 2020; Lei Xiaojun, 2021) involving 450 patients with gout evaluated the Upro levels. The results demonstrated that adding DGNT to conventional therapy has a therapeutic effect on Upro. Therefore, adding DGNT on the basis of CWM treatment can significantly reduce the Upro levels [WMD = −0.72; 95% CI (−0.91, −0.53); *p* = 0.000, random effects model; see Figure 6]. There was significant heterogeneity (I^2^ = 93.5%; see Figure 6). The subgroup analyses indicated no significant difference between subgroups of different duration, complications, and different intervention treatments (*p* for interaction = 0.15, 0.19, and 0.28, respectively) (Table 2; Supplementary Figure S1).

##### 3.4.2.4 Interleukin-8 (IL-8)

The IL-8 levels were assessed in 3 studies (Luo Jinlin and Luo, 2012; Chunxiao, 2015; Huang Juntao and Ge, 2016) including 262 patients. The difference between the groups is significant, indicating that DGNT is beneficial [WMD = −4.77; 95% CI (−11.48, 1.94); *p* = 0.000, random effects model; see Figure 6]. There was a significant heterogeneity observed (I^2^ = 88.7%; see Figure 6). Results of subgroup analyses indicated a significant difference in different intervention treatments (*p* < 0.0001), while no significant difference was observed between subgroups of different intervention duration (*p* = 0.16) (Table 2; Supplementary Figure S1).

#### 3.4.3 Adverse effects

The adverse reactions were reported in five out of 13 studies, and were mainly rash (Yue Zhao, 2019; Lei Xiaojun, 2021), nausea and vomiting (Shang, 2012; Yue Zhao, 2019; Dai Fengxiang, 2020; Guiyang, 2021; Lei Xiaojun, 2021). The total number of adverse reactions were 18 patients, including 12 in the control group and 6 in the experimental group. The adverse effects reported were neither severe nor life-threatening.

## 4 Discussion

### 4.1 Summary of the results

Compared with CWM alone, the main findings of our meta-analysis show that the combination of DGNT and CWM therapy may be more effective than CWM alone in improving BUA, CRP, ESR, IL-8, Upro, and Scr, which may require more RCT further research. Accumulating studies have shown that BUA, CRP, ESR and IL-8 play an important role in the deterioration of gout (Barros et al., 2021; Calabuig et al., 2021; Deftereos et al., 2022; Kimura et al., 2021; von Groote et al., 2021). Therefore, we reserved the above indicators in the scope of study. Although there is statistical heterogeneity among these studies, all the results suggest that compared with CWM alone, the combined use of DGNT and CWM may have a positive impact on the efficacy and safety of gout patients. Subgroup analyses were performed to explain or reduce the degree of associated heterogeneity in order to arrive at more reliable conclusions, depending on whether complications were present, different intervention times, and different types of CWMS. Among all studies, only few studies reported no side effects. In the sensitivity analysis, after the study-by-study exclusion, the results showed no significant changes and therefore the results were considered robust.

### 4.2 Quality of evidence

**FIGURE 7:**
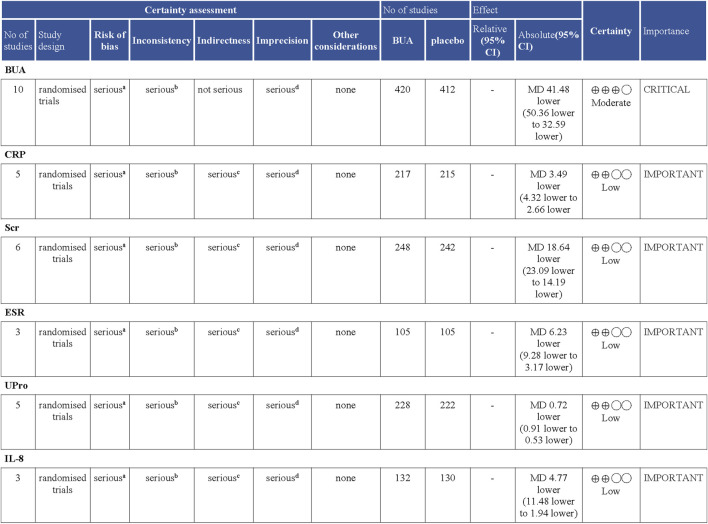
Certainty of evidence: Danggui Nianting Decoctionl compared to control treatment for gout. CI: Confidence interval; MD: Mean difference Explanations. a. Randomization allocation and the blinding are unclear in articles. b. There is serious heterogeneity among the studies included in the analysis of this outcome. c. The sample size for each indicator was less than 300 cases, and the evidence was rated down by one level. d. Results are imprecise since the study included relatively few studies and few patients.

We assessed the certainty of the evidence analyzed in this study through the GRADEpro software. As shown in Figure 7, BUA had a moderate quality of evidence, while CRP, ESR, Scr, Upro, and IL-8 had a low certainty of evidence in RCTs. The certainty of the evidence in this study is reduced for the following reasons: there is a high risk of bias in the included studies, inconsistent among the included studies, serious indirection, and inaccuracy of survey results. This is because randomization allocation and unclear blinding in articles. There was serious heterogeneity among the studies included in the analysis of this outcome, the sample size for each indicator was less than 300 cases, and the evidence was rated down by one level. Since the meta-analysis included relatively few studies and few patients, the results are imprecise. Consequently, our current findings should be considered cautiously for clinical practice. Additional and more standardized RCTs are further needed to validate the effects of DGNT on gout.

### 4.3 Strengths and limitations

To avoid confusion and ensure accuracy, we carried out this study in strict accordance with the method of systematic review, and carefully explained the results. We found that DGNT in combination with CWM might improve BUA, CRP, ESR, IL-8, Upro, and Scr in the treatment of gout. As a result, this research may provide new directions for the study of DGNT on gout.

Inevitably, there are some limitations in this meta-analysis. 1) The results of 13 included studies were all positive, all of which were single-center studies in China, and there may be some potential publication deviation risks. Therefore, results should be interpreted cautiously. 2) Small sample size and short intervention cycle (1–12 weeks) may affect the scientific and reliability of the conclusion, and it is impossible to evaluate the long-term therapeutic effect of DGNT on gout. 3) These RCTs are not so strict in experimental design and implementation, which may reduce the research quality. For example, none of the included studies described the method of randomization hidden. One study clearly pointed out the implementation of single-blind method, while other studies have not yet reported whether to implement blind method. 4) The heterogeneity in some of our results cannot be ignored. The low methodological quality of the research included in this meta-analysis may be the main reason for the high heterogeneity. This highlights the importance of strengthening the control of included research methods. In addition, as no adverse effects were reported in some studies, the safety of DGNT is unknown, and further studies are needed to confirm it. The source of heterogeneity has not been fully determined, although we have done subgroup analysis. Therefore, the clinical trials with higher quality are needed in the future.

Additionally, gout as a common endocrine metabolic disease, life interventions such as healthy diet and regular exercise are also important treatment modalities and have a great impact on the values of the biological indicators assessed. Follow-up studies could further explore the effects of different diets combined with pharmacological treatments in gout based on this study, such as the type of diet, calories, and purine content, to discuss the prevention and treatment of gout in more detail and depth. To ensure accuracy and avoid misleading conclusions, therefore, we interpret this result carefully and cautiously. This systematic review and meta-analysis described and evaluated the current clinical trials on DGNT for the treatment of gout and filled the gaps of existing knowledge. Although further studies are needed to establish the optimal approach for the application of this treatment into practice, our study adds a new possibility for the clinical management of DGNT for treating gout.

## 5 Conclusion

In all, this meta-analysis shows that DGNT in combination with CWM seems to be more effective in treating gout. In addition, the reported adverse effects were neither severe nor life-threatening. However, due to the high heterogeneity and low quality of evidence, large sample and high-quality studies are further needed to support and confirm the clinical efficacy and safety of DGNT in the treatment of gout.

## Data Availability

The original contributions presented in the study are included in the article/Supplementary Material, further inquiries can be directed to the corresponding author.

## Abbreviations

AGA, acute gouty arthritis; BUA, blood uric acid; BUN, blood urea nitrogen; CI, confidence interval; CKD, chronic kidney disease; CNKI, China National Knowledge Infrastructure; cqvip, VIP information resource integration service platform; CRE, creatinine; CRP, C-reactive protein; CVDs, cardiovascular diseases; CWM, conventional western medicine; DGNT, Danggui Niantong decoction; ESR, Erythrocyte sedimentation rate; ET-1, endothelin-1; GBD, Global Burden of Disease; GRADE, grading of recommendations assessment, development, and evaluation; IL-8, Interleukin-8; MD, mean difference; RCTs, randomized controlled trials; Scr, Serum creatinine; Sino Med, China Biology Medicine Disc; TCM, traditional Chinese medicine; Upro, Urinary protein was quantified at 24 h; WMD, weighted mean difference.
