# Supplementary material for: Efficacy and safety of Danggui Niantong Decoction in patients with gout: a systematic review and meta-analysis
Source: Front Pharmacol. 2023 Jul 26;14:1168863. doi: 10.3389/fphar.2023.1168863 (PMC10426740; doi:10.3389/fphar.2023.1168863)
Supplement: Supplementary file 1 [file DataSheet1.docx]

Supplementary Material

[Supplementary Table S1 Search strategy 2](#_Toc94122688)

[Supplementary Table S2 The composition of the prescriptions 6](#_Toc94122688)

[Supplementary Figure S1 Subgroup analysis 26](#_Toc94122690)

[Supplementary Figure S2 Sensitivity analysis 29](#_Toc94122691)

[Supplementary Figure S3 The funnel plots 30](#_Toc94122691)

Supplementary Figure S4 The PRISMA checklist of this meta-analysis..................32

#

# Supplementary Table S1 Search strategy

| **Databases** | **Search items** | **Number** |
| --- | --- | --- |
| **PubMed** | # l:"Gout" [MeSH Terms] OR"Chondrocalcinosis" [Title/ Abstract]OR "Gout Suppressants" [Title/Abstract]OR"Hypoxanthine Phosphoribosyltransferase" [Title/Abstract]  #2:"Danggui Niantong Decoction" [MeSH Terms] OR"Danggui Niantong Tang" [Title/ Abstract]OR "Danggui Niantong " [Title/Abstract]  #3:"randomized controlled trial"[Publication Type] OR "controlled clinical trial"[Publication Type] OR "randomized"[Title/Abstract] OR "placebo"[Title/Abstract] OR "randomly"[Title/Abstract] OR "trial"[Title/Abstract]OR "groups"[Title/Abstract]  #4:#1 AND #2 AND #3 | 1 |
| **Cochrane Library** | #1 MeSH descriptor: [Gout] explode all trees  #2(Chondrocalcinosis):ti,ab,kw OR (Gout Suppressants):ti,ab,kw OR (Hypoxanthine Phosphoribosyltransferase):ti,ab,kw  #3 #1 OR #2  #4 MeSH descriptor: [Danggui Niantong Decoction] explode all trees  #5 (Danggui Niantong Tang):ti,ab,kw OR (Danggui Niantong):ti,ab,kw  #6 #4 AND #5 | 0 |
| **Web of science** | #1TS=(Gout OR"Chondrocalcinosis*"OR"Gout Suppressants*"OR"Hypoxanthine Phosphoribosyltransferase*" )  #2TS=("Danggui Niantong Decoction "OR"Danggui Niantong"OR"Danggui Niantong Tang")  #3 #1 AND #2  #4TS=("randomized controlled trial"  OR"controlled clinical trial" OR "randomized*" OR "placebo*" OR"randomly" OR "trial*")  #5 #3 AND #4 | 0 |
| **Embase** | #1 'gout'/exp  #2 'chondrocalcinosis':ab,ti  #3 'gout suppressants':ab,ti  #4 'hypoxanthine phosphoribosyltransferase':ab,ti  #5 #1 OR #2 OR #3 OR #4  #6 'danggui niantong decoction'/exp  #7 'danggui niantong tang':ab,ti  #8 'danggui niantong':ab,ti  #9 #6 OR #7 OR #8  #10 'randomized controlled trial'/exp  #11 'controlled clinical trial': ti,ab OR 'randomized*':ti,ab OR 'placebo*':ti,ab OR 'randomly':ti,ab OR 'trial*': ti,ab  #12 #10 OR #11  #10 #5AND #9 AND #12 | 0 |
| **CNKI** | (SU='痛风'+'高尿酸血症'+'痛风性关节炎') AND (SU='当归拈痛汤') | 90 |
| **Wanfang Data** | (主题:("痛风" or "高尿酸血症" or "痛风性关节炎") or 题名或关键词:("痛风" or "高尿酸血症" or "痛风性关节炎") or 摘要:("痛风" or "高尿酸血症" or "痛风性关节炎")) and (主题:("当归拈痛汤") or 题名或关键词:("当归拈痛汤") or 摘要:("当归拈痛汤")) | 81 |
| **VIP** | ((M=痛风 OR 高尿酸血症 OR 痛风性关节炎) OR (K=痛风 OR 高尿酸血症 OR 痛风性关节炎) OR (R=痛风 OR 高尿酸血症 OR 痛风性关节炎)) AND ((M=当归拈痛汤) OR (K=当归拈痛汤) OR (R=当归拈痛汤)) | 62 |
| **CBM** | [("痛风"[摘要:智能] OR "高尿酸血症"[摘要:智能] OR "痛风性关节炎"[摘要:智能]) AND ("当归拈痛汤"[摘要:智能])](javascript:toDoRelimitSearch();) | 56 |

# Supplementary Table S2 The composition of the prescriptions

| **Study** | **Chinese name** | **Species, concentration** | **Quality control reported? (Y/N)** | **Chemical analysis reported? (Y/N)** |
| --- | --- | --- | --- | --- |
| Chen  (2021) | dāng guī,10g | *Angelica sinensis* (Oliv.) Diels [Umbelliferae;Angelicae Sinensis Radix],10g | N | N |
|  | qiāng huó,10g | *Notopterygium incisum* Ting ex H. T. Chang [Umbelliferae;Rhizoma Notopterygii],10g |  |  |
|  | cāng zhú,10g | *Atractylodes lancea* (Thunb.) DC.[Asteraceae; Atractylodis rhizoma],10g |  |  |
|  | bái zhú,10g | *Atractylodes macrocephala* Koidz.[Asteraceae: Atractylodis macrocephalaerhizoma],10g |  |  |
|  | zé xiè,10g | *Alisma orientalis*(Sam.)Juzep.[Alismataceae;Alismatis Rhizoma],10g |  |  |
|  | yīn chén,15g | *Artemisia capillaris* (Thunb.)DC.[Compositae;Artemisia capillaris],15g |  |  |
|  | zhī mǔ,10g | *Anemarrhena asphodeloides* Bunge. [Liliaceae;Anemarrhenae Rhizoma],10g |  |  |
|  | huáng bò,12g | *Phellodendron amurense* Rupr.[Rutaceae;CortexPhellodendriChinensis],12g |  |  |
|  | tǔ fú líng,15g | *Smilax glabra* Roxb.[Smilacaceae; Smilacis glabrae rhizoma],15g |  |  |
|  | bái máo gēn,15g | *Imperata koenigii* (Retz.) Beauv.[Gramineae; Rhizoma Imperatae],15g |  |  |
|  | wēi líng xiān,10g | *Clematis chinensis* Osbeck.[Ranunculaceae;ClematisfloridaThunb],10g |  |  |
|  | bì xiè,10g | *Dioscorea septemloba* Thunb. [Dioscoreaceae; Dioscoreae hypoglaucae rhizoma],10g |  |  |
|  | chuān niú xī ,10g | *Cyathula officinalis* Kuan[Amaranthaceae;Radix Cyathulae],10g |  |  |
| Dai etal  (2020) | dāng guī,10g | *Angelica sinensis* (Oliv.) Diels [Umbelliferae;Angelicae Sinensis Radix],10g | N | N |
|  | qiāng huó,10g | *Notopterygium incisum* Ting ex H. T. Chang [Umbelliferae;Rhizoma Notopterygii],10g |  |  |
|  | cāng zhú,20g | *Atractylodes lancea* (Thunb.) DC.[Asteraceae; Atractylodis rhizoma],20g |  |  |
|  | bái zhú,30g | *Atractylodes macrocephala* Koidz.[Asteraceae: Atractylodis macrocephalaerhizoma],30g |  |  |
|  | gé gēn,10g | *Radix Puerariae* Lobatae.[Leguminosae;Radix Puerariae],10g |  |  |
|  | yīn chén,20g | *Artemisia capillaris* (Thunb.)DC.[Compositae;Artemisia capillaris],20g |  |  |
|  | fáng fēng,10g | *Saposhnikovia divaricata* (Trucz.) Schischk.[Umbelliferae;Radix Saposhnikoviae],10g |  |  |
|  | shēnɡ má,10g | *Cimicifuga foetida* L.[Ranunculaceae;Cimicifugae Rhizoma],10 g |  |  |
|  | zé xiè,10g | *Alisma orientalis*(Sam.)Juzep.[Alismataceae;Alismatis Rhizoma],10 g |  |  |
|  | zhū líng,10g | *Polyporus umbellatus*（Pers）Fr.[polyporaceae;Polyporus],10 g |  |  |
|  | huáng qín,10g | *Scutellaria baicalensis* Georgi.[Labiatae;Scutellariae Radix],10 g |  |  |
|  | kǔ shēn,10g | *Sophora flavescens* Alt.[Leguminosae;Sophorae Flavescentis Radix],10 g |  |  |
|  | gān cǎo,10g | *Glycyrrhiza uralensis* Fisch. ex DC.[Fabaceae: Glycyrrhizae radix et rhizoma],10g |  |  |
| Huang etal  (2016) | dāng guī,6g | *Angelica sinensis* (Oliv.) Diels [Umbelliferae;Angelicae Sinensis Radix],6g | N | N |
|  | qiāng huó,15g | *Notopterygium incisum* Ting ex H. T. Chang [Umbelliferae;Rhizoma Notopterygii],15g |  |  |
|  | cāng zhú,9g | *Atractylodes lancea* (Thunb.) DC.[Asteraceae; Atractylodis rhizoma],9g |  |  |
|  | bái zhú,3g | *Atractylodes macrocephala* Koidz.[Asteraceae: Atractylodis macrocephalaerhizoma],3g |  |  |
|  | yīn chén,15g | *Artemisia capillaris* (Thunb.)DC.[Compositae;Artemisia capillaris],15g |  |  |
|  | dǎng shēn,9g | *Codonopsis pilosula* (Franch.) Nannf.[Campanulaceae; Codonopsis radix],9g |  |  |
|  | zhū líng,9g | *Polyporus umbellatus*（Pers）Fr.[polyporaceae;Polyporus],9g |  |  |
|  | zé xiè,9g | *Alisma orientalis*(Sam.)Juzep.[Alismataceae;Alismatis Rhizoma],9g |  |  |
|  | huáng qín,6g | *Scutellaria baicalensis* Georgi.[Labiatae;Scutellariae Radix],6g |  |  |
|  | zhī mǔ,9g | *Anemarrhena asphodeloides* Bunge [Liliaceae;Anemarrhenae Rhizoma],9g |  |  |
|  | fáng fēng,9g | *Saposhnikovia divaricata* (Trucz.) Schischk.[Umbelliferae;Radix Saposhnikoviae],9g |  |  |
|  | kǔ shēn,6g | *Sophora flavescens* Alt.[Leguminosae;Sophorae Flavescentis Radix],6g |  |  |
|  | shēnɡ má,9g | *Cimicifuga foetida* L.[Ranunculaceae;Cimicifugae Rhizoma],9g |  |  |
|  | gé gēn,3g | *Radix Puerariae* Lobatae.[Leguminosae;Radix Puerariae],3g |  |  |
|  | zhì gān cǎo,15g | *Glycyrrhiza uralensis* Fisch. ex DC.(Fabaceae: Glycyrrhizae radix et rhizoma],15g |  |  |
| Jiang  (2015) | dāng guī,9g | *Angelica sinensis* (Oliv.) Diels [Umbelliferae;Angelicae Sinensis Radix],9g | N | N |
|  | qiāng huó,15g | *Notopterygium incisum* Ting ex H. T. Chang [Umbelliferae;Rhizoma Notopterygii],15g |  |  |
|  | cāng zhú,9g | *Atractylodes lancea* (Thunb.) DC.[Asteraceae; Atractylodis rhizoma],9g |  |  |
|  | bái zhú,3g | *Atractylodes macrocephala* Koidz.[Asteraceae: Atractylodis macrocephalaerhizoma],3g |  |  |
|  | yīn chén,15g | *Artemisia capillaris* (Thunb.)DC.[Compositae;Artemisia capillaris],15g |  |  |
|  | zhī mǔ,9g | *Anemarrhena asphodeloides* Bunge [Liliaceae;Anemarrhenae Rhizoma],9g |  |  |
|  | zhū líng,9g | *Polyporus umbellatus*（Pers）Fr.[polyporaceae;Polyporus],9g |  |  |
|  | zé xiè,9g | *Alisma orientalis*(Sam.)Juzep.[Alismataceae;Alismatis Rhizoma],9g |  |  |
|  | fáng fēng,9g | *Saposhnikovia divaricata* (Trucz.) Schischk.[Umbelliferae;Radix Saposhnikoviae],9g |  |  |
|  | gé gēn,6g | *Radix Puerariae* Lobatae.[Leguminosae;Radix Puerariae],6g |  |  |
|  | dǎng shēn,6g | *Codonopsis pilosula* (Franch.) Nannf.[Campanulaceae; Codonopsis radix],6g |  |  |
|  | kǔ shēn,6g | *Sophora flavescens* Alt.[Leguminosae;Sophorae Flavescentis Radix],6g |  |  |
|  | huáng qín,3g | *Scutellaria baicalensis* Georgi.[Labiatae;Scutellariae Radix],3g |  |  |
|  | shēnɡ má,3g | *Cimicifuga foetida* L.[Ranunculaceae;Cimicifugae Rhizoma],3g |  |  |
|  | zhì gān cǎo,15g | *Glycyrrhiza uralensis* Fisch. ex DC.(Fabaceae: Glycyrrhizae radix et rhizoma],15g |  |  |
| Lu etal  (2021) | dāng guī,9g | *Angelica sinensis* (Oliv.) Diels [Umbelliferae;Angelicae Sinensis Radix],9g | N | N |
|  | qiāng huó,15g | *Notopterygium incisum* Ting ex H. T. Chang [Umbelliferae;Rhizoma Notopterygii],15g |  |  |
|  | cāng zhú,9g | *Atractylodes lancea* (Thunb.) DC.[Asteraceae; Atractylodis rhizoma],9g |  |  |
|  | bái zhú,3g | *Atractylodes macrocephala* Koidz.[Asteraceae: Atractylodis macrocephalaerhizoma],3g |  |  |
|  | yīn chén,15g | *Artemisia capillaris* (Thunb.)DC.[Compositae;Artemisia capillaris],15ｇ |  |  |
|  | fáng fēng,9g | *Saposhnikovia divaricata* (Trucz.) Schischk.[Umbelliferae;Radix Saposhnikoviae],9g |  |  |
|  | zhī mǔ,9g | *Anemarrhena asphodeloides* Bunge [Liliaceae;Anemarrhenae Rhizoma],9g |  |  |
|  | zhū líng,9g | *Polyporus umbellatus*（Pers）Fr.[polyporaceae;Polyporus],9g |  |  |
|  | zé xiè,9g | *Alisma orientalis*(Sam.)Juzep.[Alismataceae;Alismatis Rhizoma],9g |  |  |
|  | shēnɡ má,3g | *Cimicifuga foetida* L.[Ranunculaceae;Cimicifugae Rhizoma],3g |  |  |
|  | kǔ shēn,6g | *Sophora flavescens* Alt.[Leguminosae;Sophorae Flavescentis Radix],6g |  |  |
|  | huáng qín,3g | *Scutellaria baicalensis* Georgi.[Labiatae;Scutellariae Radix],3g |  |  |
|  | gé gēn,6g | *Radix Puerariae* Lobatae.[Leguminosae;Radix Puerariae],6g |  |  |
|  | rén shēn6g | *Panax ginseng* C. A. Mey.[Araliaceae;Ginseng Radix Et Rhizoma],6g |  |  |
|  | gān cǎo,15g | *Glycyrrhiza uralensis* Fisch. ex DC.(Fabaceae: Glycyrrhizae radix et rhizoma],15g |  |  |
| Luo etal (2012) | dāng guī,9g | *Angelica sinensis* (Oliv.) Diels [Umbelliferae;Angelicae Sinensis Radix],9g | N | N |
|  | qiāng huó,15g | *Notopterygium incisum* Ting ex H. T. Chang [Umbelliferae;Rhizoma Notopterygii],15g |  |  |
|  | cāng zhú,9g | *Atractylodes lancea* (Thunb.) DC.[Asteraceae; Atractylodis rhizoma],9g |  |  |
|  | bái zhú,3g | *Atractylodes macrocephala* Koidz.[Asteraceae: Atractylodis macrocephalaerhizoma],3g |  |  |
|  | yīn chén,15g | *Artemisia capillaris* (Thunb.)DC.[Compositae;Artemisia capillaris],15g |  |  |
|  | fáng fēng,9g | *Saposhnikovia divaricata* (Trucz.) Schischk.[Umbelliferae;Radix Saposhnikoviae],9g |  |  |
|  | gé gēn,6g | *Radix Puerariae* Lobatae.[Leguminosae;Radix Puerariae],6g |  |  |
|  | zhū líng,9g | *Polyporus umbellatus*（Pers）Fr.[polyporaceae;Polyporus],9g |  |  |
|  | zé xiè,9g | *Alisma orientalis*(Sam.)Juzep.[Alismataceae;Alismatis Rhizoma],9g |  |  |
|  | dǎng shēn,6g | *Codonopsis pilosula* (Franch.) Nannf.[Campanulaceae; Codonopsis radix]6g |  |  |
|  | shēnɡ má,3g | *Cimicifuga foetida* L.[Ranunculaceae;Cimicifugae Rhizoma],3g |  |  |
|  | kǔ shēn,6g | *Sophora flavescens* Alt.[Leguminosae;Sophorae Flavescentis Radix],6g |  |  |
|  | huáng qín,3g | *Scutellaria baicalensis* Georgi.[Labiatae;Scutellariae Radix],3g |  |  |
|  | zhī mǔ,9g | *Anemarrhena asphodeloides* Bunge [Liliaceae;Anemarrhenae Rhizoma],9g |  |  |
|  | zhì gān cǎo,15g | *Glycyrrhiza uralensis* Fisch. ex DC.(Fabaceae: Glycyrrhizae radix et rhizoma],15g |  |  |
| Luo (2020) | dāng guī,20g | *Angelica sinensis* (Oliv.) Diels [Umbelliferae;Angelicae Sinensis Radix],20g | N | N |
|  | qiāng huó,15g | *Notopterygium incisum* Ting ex H. T. Chang [Umbelliferae;Rhizoma Notopterygii], 15g |  |  |
|  | cāng zhú,15g | *Atractylodes lancea* (Thunb.) DC.[Asteraceae; Atractylodis rhizoma], 15g |  |  |
|  | bái zhú,15g | *Atractylodes macrocephala* Koidz.[Asteraceae: Atractylodis macrocephalaerhizoma], 15g |  |  |
|  | zhū líng,10g | *Polyporus umbellatus*（Pers）Fr.[polyporaceae;Polyporus], 10g |  |  |
|  | zé xiè,10g | *Alisma orientalis*(Sam.)Juzep.[Alismataceae;Alismatis Rhizoma], 10g |  |  |
|  | gé gēn,15g | *Radix Puerariae* Lobatae.[Leguminosae;Radix Puerariae], 15g |  |  |
|  | dǎng shēn,30g | *Codonopsis pilosula* (Franch.) Nannf.[Campanulaceae; Codonopsis radix] 30g |  |  |
|  | shēnɡ má,15g | *Cimicifuga foetida* L.[Ranunculaceae;Cimicifugae Rhizoma], 15g |  |  |
|  | zhī mǔ,12g | *Anemarrhena asphodeloides* Bunge [Liliaceae;Anemarrhenae Rhizoma],12g |  |  |
|  | gān cǎo,8g | *Glycyrrhiza uralensis* Fisch. ex DC.(Fabaceae: Glycyrrhizae radix et rhizoma], 8g |  |  |
|  | qín jiāo,24g | *Gentiana macrophylla* Pall.[Gentianaceae;Gentianae Macrophyllae Radix],24g |  |  |
|  | huáng bò,15g | *Phellodendron amurense* Rupr.[Rutaceae;CortexPhellodendriChinensis],15g |  |  |
|  | yì yǐ rén,20g | *Coix lacryma-jobi* L.[Poaceae; Coicis semen],20g |  |  |
|  | quán xiē,6g | *Scorpio*.[Scorpionidae;Buthus martensii Karsch],6g |  |  |
|  | dì lóng,15g | *Pheretima aspergillum*(E. Perrier).[Asteraceae;Pheretima],15g |  |  |
|  | jīn yín huā,20g | *Lonicera japonica* Thunb.[Caprifoliaceae;Lonicerae Japonicae],20g |  |  |
|  | lián qiáo,15g | *Forsythia suspensa* (Thunb.) Vahl.[Oleaceae;Forsythiae Fructus],15g |  |  |
|  | zǐ huā dì dīnɡ,15g | *Viola philipica* Cav.[V.confusa Champ.；V.yedoensis Makino.],15g |  |  |
| Qi  (2021) | dāng guī,10g | *Angelica sinensis* (Oliv.) Diels [Umbelliferae;Angelicae Sinensis Radix],10g | N | N |
|  | qiāng huó,10g | *Notopterygium incisum* Ting ex H. T. Chang [Umbelliferae;Rhizoma Notopterygii],10g |  |  |
|  | cāng zhú,20g | *Atractylodes lancea* (Thunb.) DC.[Asteraceae; Atractylodis rhizoma],20g |  |  |
|  | bái zhú,30g | *Atractylodes macrocephala* Koidz.[Asteraceae: Atractylodis macrocephalaerhizoma],30g |  |  |
|  | zhū líng,9g | *Polyporus umbellatus*（Pers）Fr.[polyporaceae;Polyporus],9g |  |  |
|  | zé xiè,9g | *Alisma orientalis*(Sam.)Juzep.[Alismataceae;Alismatis Rhizoma],9g |  |  |
|  | kǔ shēn,10g | *Sophora flavescens* Alt.[Leguminosae;Sophorae Flavescentis Radix],10g |  |  |
|  | gé gēn,10g | *Radix Puerariae* Lobatae.[Leguminosae;Radix Puerariae],10g |  |  |
|  | fáng fēng,9g | *Saposhnikovia divaricata* (Trucz.) Schischk.[Umbelliferae;Radix Saposhnikoviae],9g |  |  |
|  | shēnɡ má,10g | *Cimicifuga foetida* L.[Ranunculaceae;Cimicifugae Rhizoma],10g |  |  |
|  | huáng qín,10g | *Scutellaria baicalensis* Georgi.[Labiatae;Scutellariae Radix],10g |  |  |
|  | yīn chén,20g | *Artemisia capillaris* (Thunb.)DC.[Compositae;Artemisia capillaris],20g |  |  |
|  | gān cǎo,10g | *Glycyrrhiza uralensis* Fisch. ex DC.(Fabaceae: Glycyrrhizae radix et rhizoma],10g |  |  |
| Sun (2020) | dāng guī,8g | *Angelica sinensis* (Oliv.) Diels [Umbelliferae;Angelicae Sinensis Radix],8g | N | N |
|  | qiāng huó,15g | *Notopterygium incisum* Ting ex H. T. Chang [Umbelliferae;Rhizoma Notopterygii],15g |  |  |
|  | cāng zhú,10g | *Atractylodes lancea* (Thunb.) DC.[Asteraceae; Atractylodis rhizoma],10g |  |  |
|  | bái zhú,10g | *Atractylodes macrocephala* Koidz.[Asteraceae: Atractylodis macrocephalaerhizoma],10g |  |  |
|  | yīn chén,15g | *Artemisia capillaris* (Thunb.)DC.[Compositae;Artemisia capillaris],15g |  |  |
|  | dān shēn,10g | *Salvia miltiorrhiza* Bunge [Lamiaceae; Salviae miltiorrhizae radix et rhizoma],10g |  |  |
|  | fáng fēng,10g | *Saposhnikovia divaricata* (Trucz.) Schischk.[Umbelliferae;Radix Saposhnikoviae],10g |  |  |
|  | zé xiè,10g | *Alisma orientalis*(Sam.)Juzep.[Alismataceae;Alismatis Rhizoma],10g |  |  |
|  | kǔ shēn,8g | *Sophora flavescens* Alt.[Leguminosae;Sophorae Flavescentis Radix],8g |  |  |
|  | gān cǎo,15g | *Glycyrrhiza uralensis* Fisch. ex DC.(Fabaceae: Glycyrrhizae radix et rhizoma],15g |  |  |
|  | bì xiè,15g | *Dioscorea septemloba* Thunb. [Dioscoreaceae; Dioscoreae hypoglaucae rhizoma],15g |  |  |
|  | dà huáng,8g | *Rheum palmatum* L.[Polygonaceae; Rhei radix et rhizomal],8g |  |  |
|  | hǔ zhàng ,8g | *Reynoutria japonica* Houtt.[Polygonaceae;Polygonum cuspidatum Sieb.et Zucc],8g |  |  |
| Zhao etal  (2019) | dāng guī,9g | *Angelica sinensis* (Oliv.) Diels [Umbelliferae;Angelicae Sinensis Radix],9g | N | N |
|  | qiāng huó,15g | *Notopterygium incisum* Ting ex H. T. Chang [Umbelliferae;Rhizoma Notopterygii],15g |  |  |
|  | cāng zhú,10g | *Atractylodes lancea* (Thunb.) DC.[Asteraceae; Atractylodis rhizoma],10g |  |  |
|  | bái zhú,10g | *Atractylodes macrocephala* Koidz.[Asteraceae: Atractylodis macrocephalaerhizoma],10g |  |  |
|  | zé xiè,9g | *Alisma orientalis*(Sam.)Juzep.[Alismataceae;Alismatis Rhizoma],9g |  |  |
|  | zhū líng,9g | *Polyporus umbellatus*(Pers)Fr.[polyporaceae;Polyporus],9g |  |  |
|  | yīn chén,15g | *Artemisia capillaris* (Thunb.)DC.[Compositae;Artemisia capillaris],15g |  |  |
|  | fáng fēng,9g | *Saposhnikovia divaricata* (Trucz.) Schischk.[Umbelliferae;Radix Saposhnikoviae],9g |  |  |
|  | kǔ shēn,6g | *Sophora flavescens* Alt.[Leguminosae;Sophorae Flavescentis Radix],6g |  |  |
|  | gān cǎo,6g | *Glycyrrhiza uralensis* Fisch. ex DC.(Fabaceae: Glycyrrhizae radix et rhizoma],6g |  |  |
|  | dà huáng,8g | *Rheum palmatum* L.[Polygonaceae; Rhei radix et rhizomal],8g |  |  |
|  | dān shēn,10g | *Salvia miltiorrhiza* Bunge [Lamiaceae; Salviae miltiorrhizae radix et rhizoma],10g |  |  |
|  | hǔ zhàng ,20g | *Reynoutria japonica* Houtt.[Polygonaceae;Polygonum cuspidatum Sieb.et Zucc],20g |  |  |
|  | bì xiè,15g | *Dioscorea septemloba* Thunb. [Dioscoreaceae; Dioscoreae hypoglaucae rhizoma],15g |  |  |
| Liu  (2018) | dāng guī,15g | *Angelica sinensis* (Oliv.) Diels [Umbelliferae;Angelicae Sinensis Radix],15g | N | N |
|  | qiāng huó,25g | *Notopterygium incisum* Ting ex H. T. Chang [Umbelliferae;Rhizoma Notopterygii],25g |  |  |
|  | cāng zhú,15g | *Atractylodes lancea* (Thunb.) DC.[Asteraceae; Atractylodis rhizoma],15g |  |  |
|  | bái zhú,5g | *Atractylodes macrocephala* Koidz.[Asteraceae: Atractylodis macrocephalaerhizoma],5g |  |  |
|  | zhū líng,15g | *Polyporus umbellatus*（Pers）Fr.[polyporaceae;Polyporus],15g |  |  |
|  | zé xiè,15g | *Alisma orientalis*(Sam.)Juzep.[Alismataceae;Alismatis Rhizoma],15g |  |  |
|  | yīn chén,25g | *Artemisia capillaris* (Thunb.)DC.[Compositae;Artemisia capillaris],25g |  |  |
|  | huáng qín,5g | *Scutellaria baicalensis* Georgi.[Labiatae;Scutellariae Radix],5g |  |  |
|  | zhī mǔ,15g | *Anemarrhena asphodeloides* Bunge [Liliaceae;Anemarrhenae Rhizoma],15g |  |  |
|  | gé gēn,10g | *Radix Puerariae* Lobatae.[Leguminosae;Radix Puerariae],10g |  |  |
|  | rén shēn,10g | *Panax ginseng* C. A. Mey.[Araliaceae;Ginseng Radix Et Rhizoma]10g |  |  |
|  | kǔ shēn,10g | *Sophora flavescens* Alt.[Leguminosae;Sophorae Flavescentis Radix],10g |  |  |
|  | shēnɡ má,5g | *Cimicifuga foetida* L.[Ranunculaceae;Cimicifugae Rhizoma],5g |  |  |
|  | fáng fēng,15g | *Saposhnikovia divaricata* (Trucz.) Schischk.[Umbelliferae;Radix Saposhnikoviae],15g |  |  |
|  | gān cǎo,25g | *Glycyrrhiza uralensis* Fisch. ex DC.(Fabaceae: Glycyrrhizae radix et rhizoma],25g |  |  |
| Lei etal  (2021) | dāng guī,9g | *Angelica sinensis* (Oliv.) Diels [Umbelliferae;Angelicae Sinensis Radix],9g | N | N |
|  | qiāng huó,15g | *Notopterygium incisum* Ting ex H. T. Chang [Umbelliferae;Rhizoma Notopterygii],15g |  |  |
|  | cāng zhú,10g | *Atractylodes lancea* (Thunb.) DC.[Asteraceae; Atractylodis rhizoma],10g |  |  |
|  | bái zhú,10g | *Atractylodes macrocephala* Koidz.[Asteraceae: Atractylodis macrocephalaerhizoma],10g |  |  |
|  | zhū líng,9g | *Polyporus umbellatus*（Pers）Fr.[polyporaceae;Polyporus],9g |  |  |
|  | zé xiè,6g | *Alisma orientalis*(Sam.)Juzep.[Alismataceae;Alismatis Rhizoma],6g |  |  |
|  | fáng fēng,15g | *Saposhnikovia divaricata* (Trucz.) Schischk.[Umbelliferae;Radix Saposhnikoviae],15g |  |  |
|  | zhī mǔ,10g | *Anemarrhena asphodeloides* Bunge [Liliaceae;Anemarrhenae Rhizoma],10g |  |  |
|  | yīn chén,10g | *Artemisia capillaris* (Thunb.)DC.[Compositae;Artemisia capillaris],10g |  |  |
|  | kǔ shēn,8g | *Sophora flavescens* Alt.[Leguminosae;Sophorae Flavescentis Radix],8g |  |  |
|  | gé gēn,6g | *Radix Puerariae* Lobatae.[Leguminosae;Radix Puerariae],6g |  |  |
|  | shēnɡ má,6g | *Cimicifuga foetida* L.[Ranunculaceae;Cimicifugae Rhizoma],6g |  |  |
|  | rén shēn,3g | *Panax ginseng* C. A. Mey.[Araliaceae;Ginseng Radix Et Rhizoma],3g |  |  |
|  | gān cǎo,10g | *Glycyrrhiza uralensis* Fisch. ex DC.(Fabaceae: Glycyrrhizae radix et rhizoma],10g |  |  |
|  | hǔ zhàng ,15g | *Reynoutria japonica* Houtt.[Polygonaceae;Polygonum cuspidatum Sieb.et Zucc],15g |  |  |
|  | bì xiè,15g | *Dioscorea septemloba* Thunb. [Dioscoreaceae; Dioscoreae hypoglaucae rhizoma],15g |  |  |
|  | dān shēn,15g | *Salvia miltiorrhiza* Bunge [Lamiaceae; Salviae miltiorrhizae radix et rhizoma],15g |  |  |
|  | dà huáng,9g | *Rheum palmatum* L.[Polygonaceae; Rhei radix et rhizomal],9g |  |  |
| Huang  (2012) | dāng guī,9g | *Angelica sinensis* (Oliv.) Diels [Umbelliferae;Angelicae Sinensis Radix],9g | N | N |
|  | qiāng huó,15g | *Notopterygium incisum* Ting ex H. T. Chang [Umbelliferae;Rhizoma Notopterygii],15g |  |  |
|  | cāng zhú,10g | *Atractylodes lancea* (Thunb.) DC.[Asteraceae; Atractylodis rhizoma],10g |  |  |
|  | bái zhú,10g | *Atractylodes macrocephala* Koidz.[Asteraceae: Atractylodis macrocephalaerhizoma],10g |  |  |
|  | zhū líng,9g | *Polyporus umbellatus*（Pers）Fr.[polyporaceae;Polyporus],9g |  |  |
|  | zé xiè,9g | *Alisma orientalis*(Sam.)Juzep.[Alismataceae;Alismatis Rhizoma],9g |  |  |
|  | yīn chén,15g | *Artemisia capillaris* (Thunb.)DC.[Compositae;Artemisia capillaris],15g |  |  |
|  | kǔ shēn,6g | *Sophora flavescens* Alt.[Leguminosae;Sophorae Flavescentis Radix],6g |  |  |
|  | gān cǎo,6g | *Glycyrrhiza uralensis* Fisch. ex DC.(Fabaceae: Glycyrrhizae radix et rhizoma],6g |  |  |
|  | bì xiè,15g | *Dioscorea septemloba* Thunb. [Dioscoreaceae; Dioscoreae hypoglaucae rhizoma],15g |  |  |
|  | dà huáng,8g | *Rheum palmatum* L.[Polygonaceae; Rhei radix et rhizomal],8g |  |  |
|  | Hǔ zhàng ,20g | *Reynoutria japonica* Houtt.[Polygonaceae;Polygonum cuspidatum Sieb.et Zucc],20g |  |  |
|  | dān shēn,10g | *Salvia miltiorrhiza* Bunge [Lamiaceae; Salviae miltiorrhizae radix et rhizoma],10g |  |  |

Supplementary Figure S1 Subgroup analysis

1. Subgroup analysis for BUA.


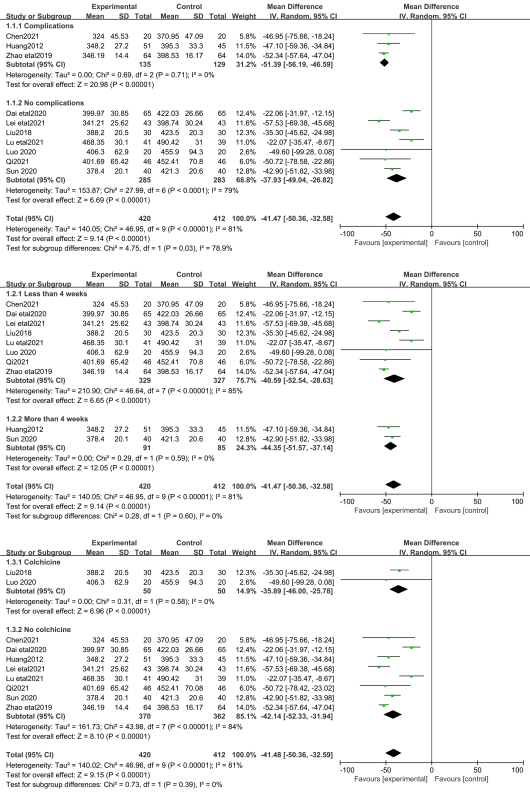


1. Subgroup analysis for CRP.


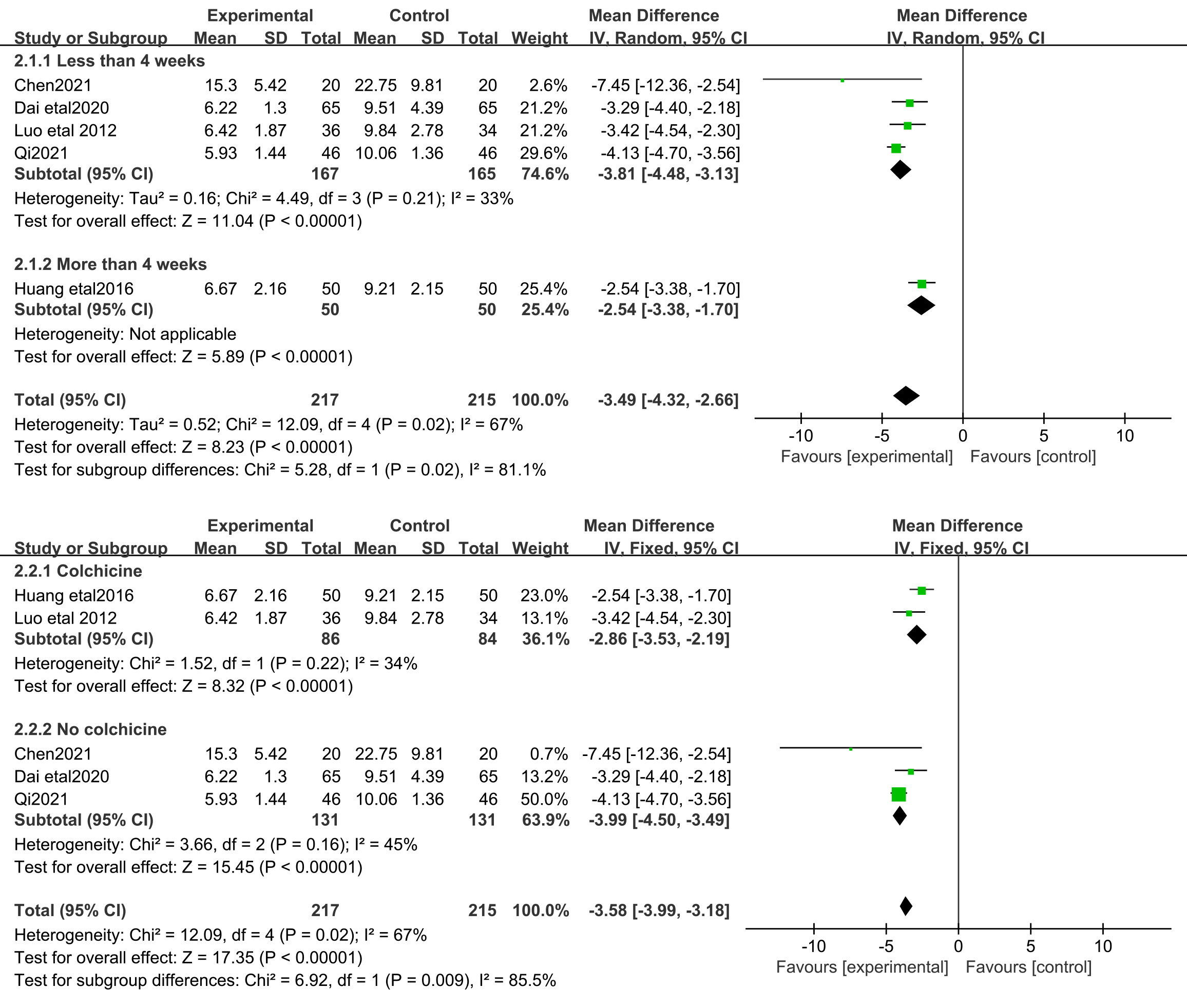


1. Subgroup analysis for ESR.


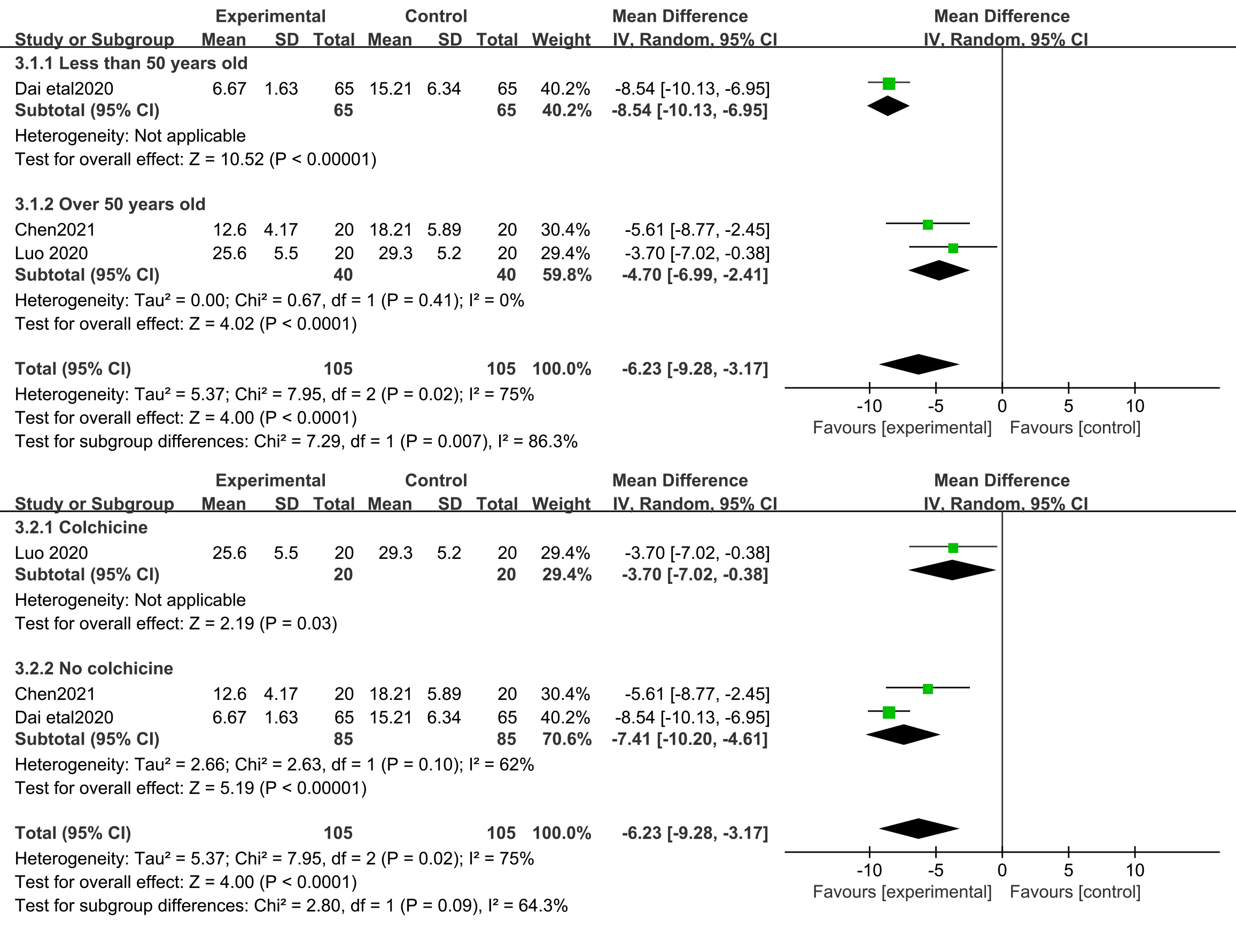


1. Subgroup analysis for Scr.


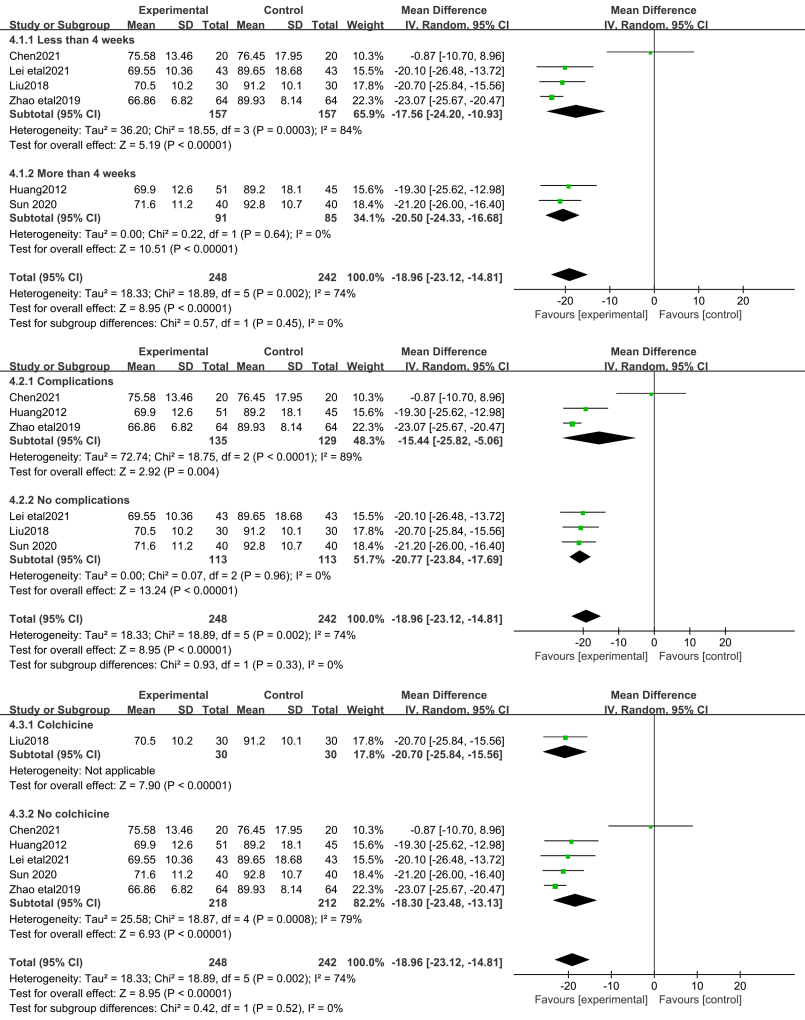


1. Subgroup analysis for Upro.


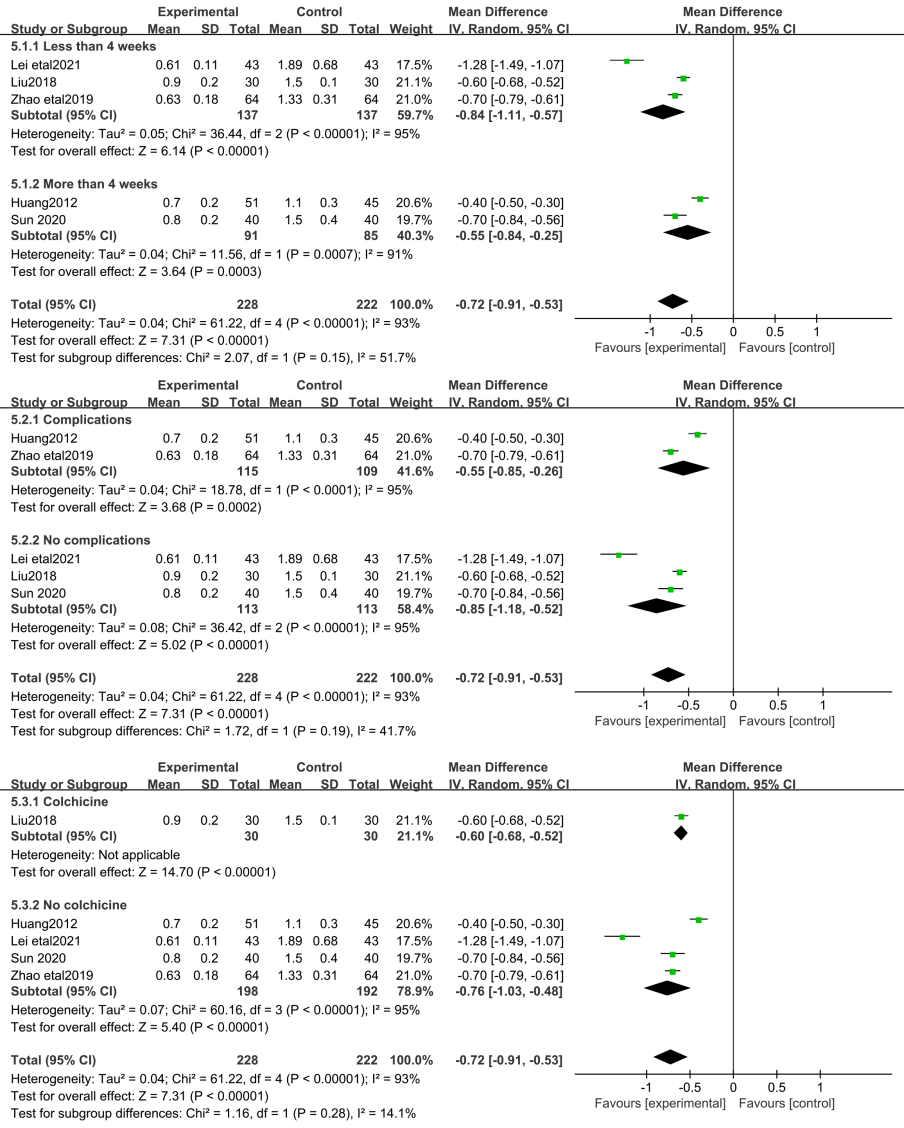


1. Subgroup analysis for IL-8.


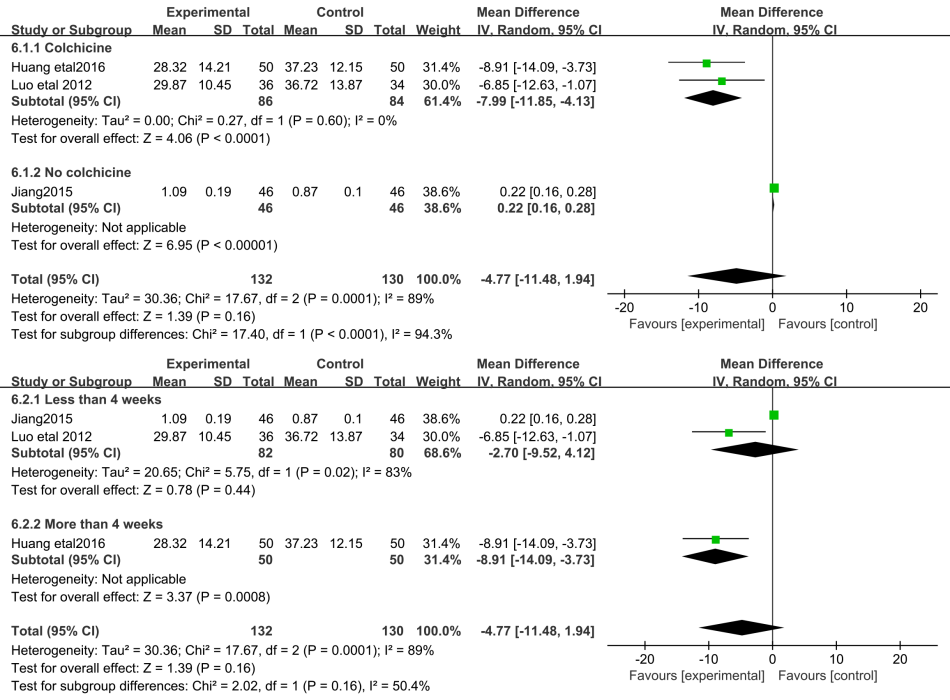


# Supplementary Figure S2 Sensitivity analysis

1.Sensitivity analysis for BUA,CRP,Scr and Upro.


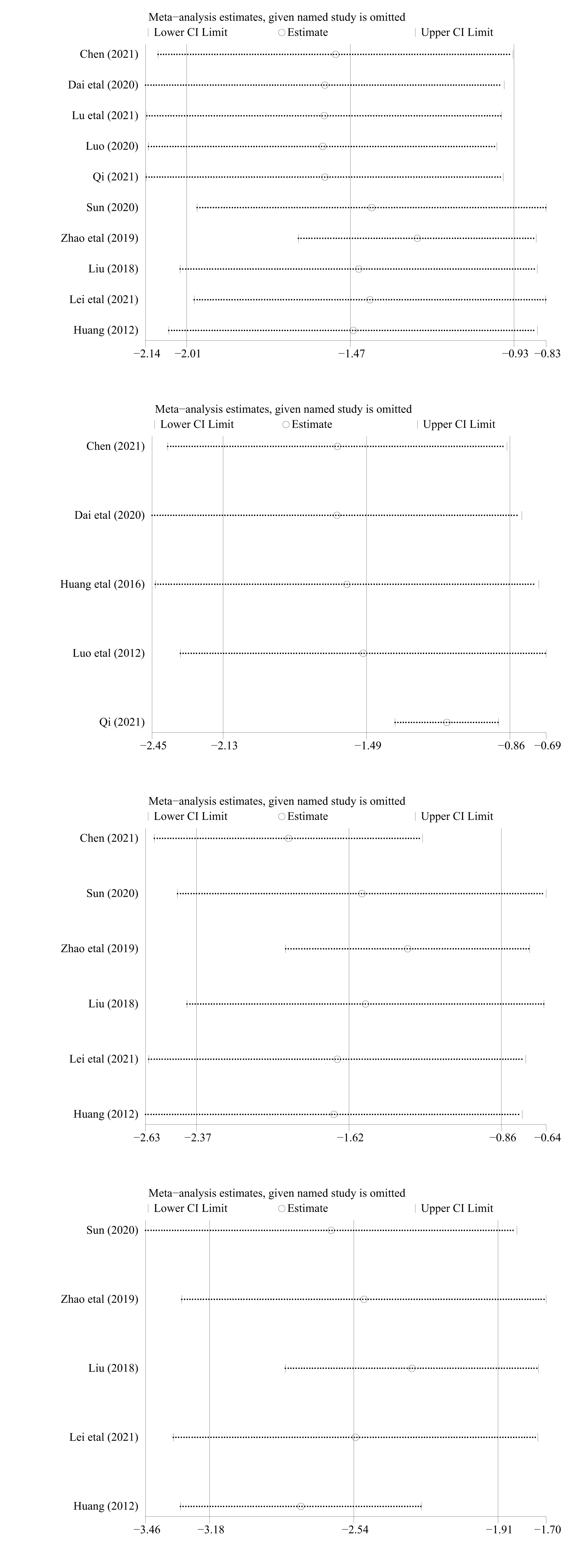


Supplementary Figure S3 The funnel plots for assessing publication bias

1.The funnel plots for BUA,CRP,ESR.


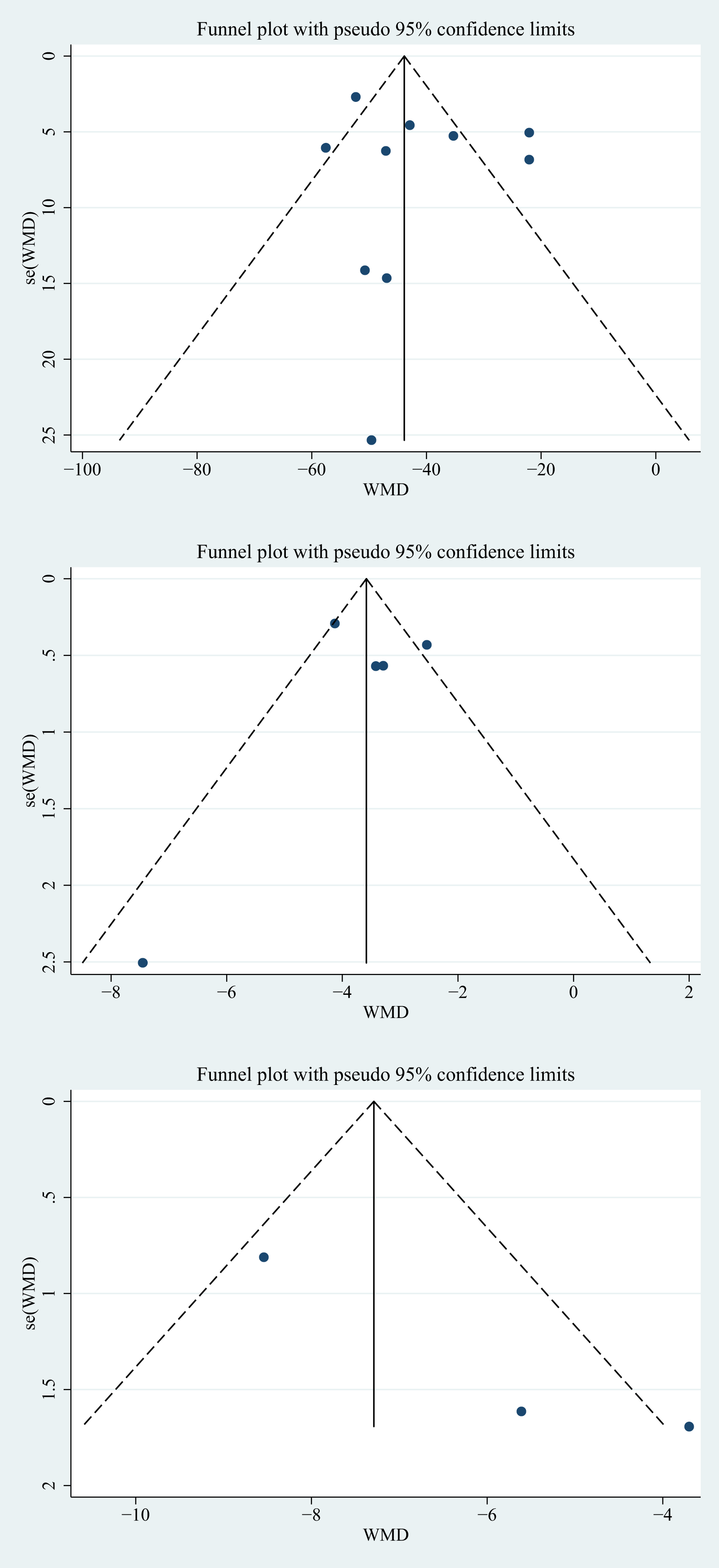


2.The funnel plots for Scr,Upro and IL-8.


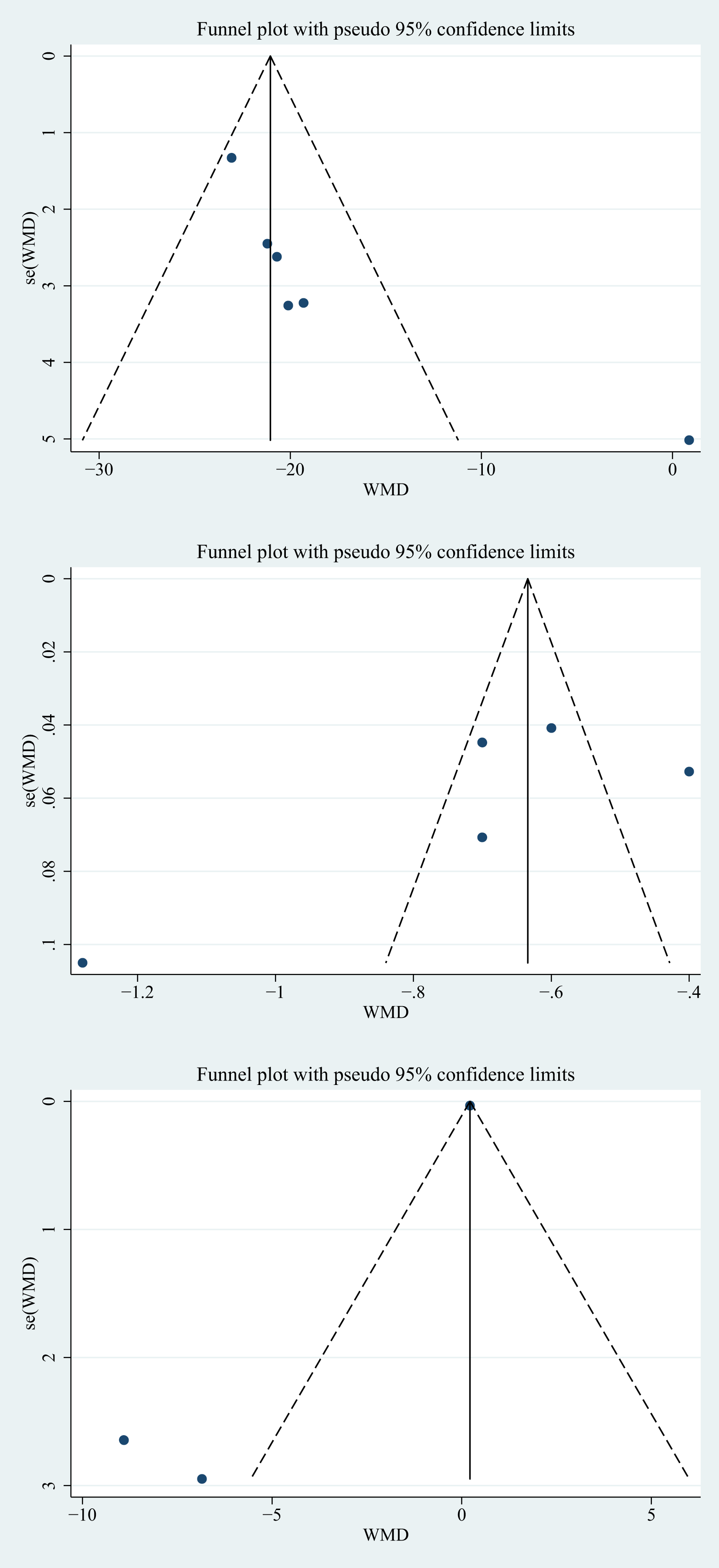


# Supplementary File S4 The PRISMA checklist of this meta-analysis

| **Section/topic** | | **#** | | **Checklist item** | | **Reported on page #** | |
| --- | --- | --- | --- | --- | --- | --- | --- |
| **TITLE** | | | | | |  | |
| Title | | 1 | | Identify the report as a systematic review, meta-analysis, or both. | | 1 | |
| **ABSTRACT** | | | | | |  | |
| Structured summary | | 2 | | Provide a structured summary including, as applicable: background; objectives; data sources; study eligibility criteria, participants, and interventions; study appraisal and synthesis methods; results; limitations; conclusions and implications of key findings; systematic review registration number. | | 1-2 | |
| **INTRODUCTION** | | | | | |  | |
| Rationale | | 3 | | Describe the rationale for the review in the context of what is already known. | | 2-3 | |
| Objectives | | 4 | | Provide an explicit statement of questions being addressed with reference to participants, interventions, comparisons, outcomes, and study design (PICOS). | | 3 | |
| **METHODS** | | | | | |  | |
| Protocol and registration | | 5 | | Indicate if a review protocol exists, if and where it can be accessed (e.g., Web address), and, if available, provide registration information including registration number. | | 3;  CRD42021271607 | |
| Eligibility criteria | | 6 | | Specify study characteristics (e.g., PICOS, length of follow-up) and report characteristics (e.g., years considered, language, publication status) used as criteria for eligibility, giving rationale. | | 4 | |
| Information sources | | 7 | | Describe all information sources (e.g., databases with dates of coverage, contact with study authors to identify additional studies) in the search and date last searched. | | 3 | |
| Search | | 8 | | Present full electronic search strategy for at least one database, including any limits used, such that it could be repeated. | | 3; Supplement Table S1 | |
| Study selection | | 9 | | State the process for selecting studies (i.e., screening, eligibility, included in systematic review, and, if applicable, included in the meta-analysis). | | 4 | |
| Data collection process | | 10 | | Describe method of data extraction from reports (e.g., piloted forms, independently, in duplicate) and any processes for obtaining and confirming data from investigators. | | 4-5 | |
| Data items | | 11 | | List and define all variables for which data were sought (e.g., PICOS, funding sources) and any assumptions and simplifications made. | | 4-5 | |
| Risk of bias in individual studies | | 12 | | Describe methods used for assessing risk of bias of individual studies (including specification of whether this was done at the study or outcome level), and how this information is to be used in any data synthesis. | | 4 | |
| Summary measures | | 13 | | State the principal summary measures (e.g., risk ratio, difference in means). | | 4-5 | |
| Synthesis of results | | 14 | | Describe the methods of handling data and combining results of studies, if done, including measures of consistency (e.g., I^2^) for each meta-analysis. | | 4-5 | |
| Section/topic | | # | | Checklist item | | Reported on page # | |
| Risk of bias across studies | | 15 | | Specify any assessment of risk of bias that may affect the cumulative evidence (e.g., publication bias, selective reporting within studies). | | 4 | |
| Additional analyses | | 16 | | Describe methods of additional analyses (e.g., sensitivity or subgroup analyses, meta-regression), if done, indicating which were pre-specified. | | 4-5 | |
| **RESULTS** | | | | | |  | |
| Study selection | | 17 | | Give numbers of studies screened, assessed for eligibility, and included in the review, with reasons for exclusions at each stage, ideally with a flow diagram. | | 5; Figure1 | |
| Study characteristics | | 18 | | For each study, present characteristics for which data were extracted (e.g., study size, PICOS, follow-up period) and provide the citations. | | 5; Table2 | |
| Risk of bias within studies | | 19 | | Present data on risk of bias of each study and, if available, any outcome level assessment (see item 12). | | 6; Figure2-3 | |
| Results of individual studies | | 20 | | For all outcomes considered (benefits or harms), present, for each study: (a) simple summary data for each intervention group (b) effect estimates and confidence intervals, ideally with a forest plot. | | 6; Figure4-5 | |
| Synthesis of results | | 21 | | Present results of each meta-analysis done, including confidence intervals and measures of consistency. | | 6-8 | |
| Risk of bias across studies | | 22 | | Present results of any assessment of risk of bias across studies (see Item 15). | | 6; Figure6 | |
| Additional analysis | | 23 | | Give results of additional analyses, if done (e.g., sensitivity or subgroup analyses, meta-regression [see Item 16]). | | 6-8 | |
| **DISCUSSION** | | | | | |  | |
| Summary of evidence | | 24 | | Summarize the main findings including the strength of evidence for each main outcome; consider their relevance to key groups (e.g., healthcare providers, users, and policy makers). | | 8 | |
| Limitations | | 25 | | Discuss limitations at study and outcome level (e.g., risk of bias), and at review-level (e.g., incomplete retrieval of identified research, reporting bias). | | 8-9 | |
| Conclusions | | 26 | | Provide a general interpretation of the results in the context of other evidence, and implications for future research. | | 9 | |
| **FUNDING** | | | | | |  | |
| Funding | | 27 | | Describe sources of funding for the systematic review and other support (e.g., supply of data); role of funders for the systematic review. | | 9 | |

*From:*  Moher D, Liberati A, Tetzlaff J, Altman DG, The PRISMA Group (2009). Preferred Reporting Items for Systematic Reviews and Meta-Analyses: The PRISMA Statement. PLoS Med 6(6): e1000097. doi:10.1371/journal.pmed1000097

For more information, visit: **www.prisma-statement.org**
